# Predictive blood biomarkers and brain changes associated with age-related cognitive decline

**DOI:** 10.1093/braincomms/fcad113

**Published:** 2023-04-06

**Authors:** Tyler S Saunders, Francesca E Pozzolo, Amanda Heslegrave, Declan King, Robert I McGeachan, Maxwell P Spires-Jones, Sarah E Harris, Craig Ritchie, Graciela Muniz-Terrera, Ian J Deary, Simon R Cox, Henrik Zetterberg, Tara L Spires-Jones

**Affiliations:** UK Dementia Research Institute and Centre for Discovery Brain Sciences at the University of Edinburgh, Edinburgh, EH8 9JZ, UK; Edinburgh Dementia Prevention & Centre for Clinical Brain Sciences, University of Edinburgh, Edinburgh, EH4 2XU, UK; UK Dementia Research Institute and Centre for Discovery Brain Sciences at the University of Edinburgh, Edinburgh, EH8 9JZ, UK; United Kingdom UK Dementia Research Institute at University College London, UCL Institute of Neurology, Queen Square, London, WC1N 3BG, UK; Department of Neurodegenerative Disease, UCL Institute of Neurology, Queen Square, London, WC1N 3BG, UK; UK Dementia Research Institute and Centre for Discovery Brain Sciences at the University of Edinburgh, Edinburgh, EH8 9JZ, UK; UK Dementia Research Institute and Centre for Discovery Brain Sciences at the University of Edinburgh, Edinburgh, EH8 9JZ, UK; UK Dementia Research Institute and Centre for Discovery Brain Sciences at the University of Edinburgh, Edinburgh, EH8 9JZ, UK; Lothian Birth Cohort studies, Department of Psychology, University of Edinburgh, Edinburgh, EH8 9AD, UK; Edinburgh Dementia Prevention & Centre for Clinical Brain Sciences, University of Edinburgh, Edinburgh, EH4 2XU, UK; Edinburgh Dementia Prevention & Centre for Clinical Brain Sciences, University of Edinburgh, Edinburgh, EH4 2XU, UK; Department of Social Medicine, Ohio University, Athens, Ohio 45701, USA; Latin American Institute for Brain Health (BrainLat), Universidad Adolfo Ibanez, Santiago 3485, Chile; Lothian Birth Cohort studies, Department of Psychology, University of Edinburgh, Edinburgh, EH8 9AD, UK; Lothian Birth Cohort studies, Department of Psychology, University of Edinburgh, Edinburgh, EH8 9AD, UK; United Kingdom UK Dementia Research Institute at University College London, UCL Institute of Neurology, Queen Square, London, WC1N 3BG, UK; Department of Neurodegenerative Disease, UCL Institute of Neurology, Queen Square, London, WC1N 3BG, UK; Department of Psychiatry and Neurochemistry, Sahlgrenska Academy at the University of Gothenburg, S-431 80 Molndal, Sweden; Clinical Neurochemistry Laboratory, Sahlgrenska University Hospital, S-431 80 Molndal, Sweden; Hong Kong Center for Neurodegenerative Diseases, Clear Water Bay, Hong Kong, China; UK Dementia Research Institute and Centre for Discovery Brain Sciences at the University of Edinburgh, Edinburgh, EH8 9JZ, UK

**Keywords:** array tomography, cognition, cognitive ageing, plasma biomarkers, western blotting

## Abstract

Growing evidence supports the use of plasma levels of tau phosphorylated at threonine 181, amyloid-β, neurofilament light and glial fibrillary acidic protein as promising biomarkers for Alzheimer’s disease. While these blood biomarkers are promising for distinguishing people with Alzheimer’s disease from healthy controls, their predictive validity for age-related cognitive decline without dementia remains unclear. Further, while tau phosphorylated at threonine 181 is a promising biomarker, the distribution of this phospho-epitope of tau in the brain is unknown. Here, we tested whether plasma levels of tau phosphorylated at threonine 181, amyloid-β, neurofilament light and fibrillary acidic protein predict cognitive decline between ages 72 and 82 in 195 participants in the Lothian birth cohorts 1936 study of cognitive ageing. We further examined post-mortem brain samples from temporal cortex to determine the distribution of tau phosphorylated at threonine 181 in the brain. Several forms of tau phosphorylated at threonine 181 have been shown to contribute to synapse degeneration in Alzheimer’s disease, which correlates closely with cognitive decline in this form of dementia, but to date, there have not been investigations of whether tau phosphorylated at threonine 181 is found in synapses in Alzheimer’s disease or healthy ageing brain. It was also previously unclear whether tau phosphorylated at threonine 181 accumulated in dystrophic neurites around plaques, which could contribute to tau leakage to the periphery due to impaired membrane integrity in dystrophies. Brain homogenate and biochemically enriched synaptic fractions were examined with western blot to examine tau phosphorylated at threonine 181 levels between groups (*n* = 10–12 per group), and synaptic and astrocytic localization of tau phosphorylated at threonine 181 were examined using array tomography (*n* = 6–15 per group), and localization of tau phosphorylated at threonine 181 in plaque-associated dystrophic neurites with associated gliosis were examined with standard immunofluorescence (*n* = 8–9 per group).

Elevated baseline plasma tau phosphorylated at threonine 181, neurofilament light and fibrillary acidic protein predicted steeper general cognitive decline during ageing. Further, increasing tau phosphorylated at threonine 181 over time predicted general cognitive decline in females only. Change in plasma tau phosphorylated at threonine 181 remained a significant predictor of *g* factor decline when taking into account Alzheimer’s disease polygenic risk score, indicating that the increase of blood tau phosphorylated at threonine 181 in this cohort was not only due to incipient Alzheimer’s disease. Tau phosphorylated at threonine 181 was observed in synapses and astrocytes in both healthy ageing and Alzheimer’s disease brain. We observed that a significantly higher proportion of synapses contain tau phosphorylated at threonine 181 in Alzheimer’s disease relative to aged controls. Aged controls with pre-morbid lifetime cognitive resilience had significantly more tau phosphorylated at threonine 181 in fibrillary acidic protein-positive astrocytes than those with pre-morbid lifetime cognitive decline. Further, tau phosphorylated at threonine 181 was found in dystrophic neurites around plaques and in some neurofibrillary tangles. The presence of tau phosphorylated at threonine 181 in plaque-associated dystrophies may be a source of leakage of tau out of neurons that eventually enters the blood. Together, these data indicate that plasma tau phosphorylated at threonine 181, neurofilament light and fibrillary acidic protein may be useful biomarkers of age-related cognitive decline, and that efficient clearance of tau phosphorylated at threonine 181 by astrocytes may promote cognitive resilience.

## Teaser

Saunders *et al*. report that plasma levels of phospho-tau181, neurofilament-light and glial fibrillary acid protein predict steeper general cognitive decline in adults aged between 72 and 82 years. Further, they report the presence of phospho-tau181 in synapses and astrocytes of Alzheimer’s cases and aged controls.

## Graphical Abstract

**Graphical Abstract:**
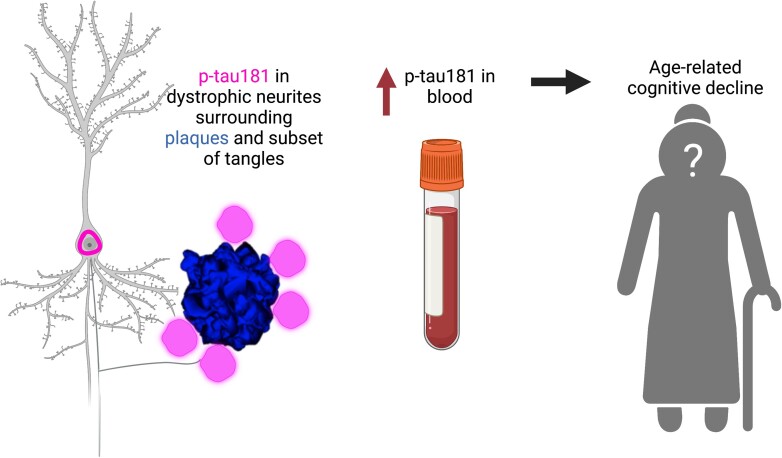

See Saura and Parra-Damas (https://doi.org/10.1093/braincomms/fcad142) for a scientific commentary on this article.

## Introduction

By the year 2050, the world’s population of people aged over 60 is expected to reach 2 billion.^1^

An ageing population will lead to an increase in the prevalence of age-related cognitive decline, which can seriously limit an individual’s independence and quality of life.^2,3^ Investigating biomarkers associated with age-related cognitive decline will improve our understanding of the underlying pathophysiology,^4^ which may eventually improve prognoses via the identification of at-risk individuals and the development of novel therapeutics. However, examining age-related changes *in vivo* via cerebrospinal fluid (CSF) analyses, positron emission tomography (PET) and magnetic resonance imaging (MRI) can be invasive and expensive. Blood-based biomarkers provide the benefits of being accessible in primary care settings and less invasive.

There is a growing interest in several plasma biomarkers associated with cognitive function, in both ageing and disease. Several pathological changes in the brain, typically seen in Alzheimer’s disease (AD) can also be observed in late-adulthood individuals without dementia. Post-mortem studies of those without dementia report the accumulation of hyperphosphorylated tau into neurofibrillary tangles, the build-up of amyloid-beta (Aβ) into plaques^5,6^ and the presence of GFAP (reflecting neuroinflammation).^6,7^ Biomarkers reflecting the pathological hallmarks of Alzheimer’s disease^8-10^ such as plasma phosphorylated tau 181 (p-tau181) and plasma Aβ (both Aβ40, Aβ42 and the Aβ42/40 ratio) have been investigated as biomarkers associated with cognitive function. Plasma p-tau181 levels are elevated in AD relative to controls,^11,12^ and studies report a negative correlation with cognitive function across the Alzheimer’s-spectrum.^12–23^ However, in sub-samples of only cognitively unimpaired individuals, many of these studies report no significant association with cognitive function.^13,16,19,21,22^ Two longitudinal studies have reported a significant negative association between plasma p-tau181 and age-related cognitive decline over 5 years.^18,23^ The ratio between Aβ42/40 and plasma p-tau181 has also been found to correlate with cognitive decline over an 8-year period in cognitively unimpaired participants aged 65 years.^24^ Another study reported a significant negative correlation between baseline p-tau181 and longitudinal grey matter volume change in cognitively unimpaired participants aged 74 years.^25^ Plasma Aβ42/40 is reported to be decreased in Alzheimer’s disease, mild cognitive impairment and subjective cognitive decline relative to those with no cognitive impairment.^26–30^ A lower Aβ42/40 ratio has been found to be associated with faster cortical Aβ accumulation,^31^ and poorer cognitive outcomes across the Alzheimer’s-spectrum^32-37^ and in cognitively unimpaired middle-to-late life individuals,^38-40^ although not all studies replicate this finding.^26,41,42^

In addition to the pathological hallmarks of Alzheimer’s disease, markers of neuronal injury such as neurofilament-light (NfL) have also been investigated as markers associated with cognitive decline. Plasma NfL is elevated across the Alzheimer’s spectrum,^12,43-46^ other neurodegenerative diseases such as frontotemporal dementia^47,48^ and has been found to increase with age.^49–51^ Plasma NfL has been found to correlate with both cross-sectional and longitudinal decline in cognitive functions in Alzheimer’s disease, mild cognitive impairment and subjective cognitive decline.^12,43,45,46,48,50,52-54^ In cognitively unimpaired individuals, some studies report a significant negative association between plasma NfL and cross-sectional cognitive functions,^12,55-57^ although not all studies replicate this finding.^53,58,59^ Studies investigating longitudinal cognitive decline and plasma NfL have also reported a significant inverse association in cognitively unimpaired participants.^12,45,56-58,60-64^

Lastly, plasma glial fibrillary acidic protein (GFAP), a marker of astrogliosis,^65,66^ is elevated across the Alzheimer’s spectrum,^67-71^ as well as other neurodegenerative diseases such as frontotemporal dementia^70,72^ and studies report a significant correlation with cognitive functioning.^37,62,68,70,72-74^ Plasma GFAP has also been reported to increase over time in cognitively unimpaired individuals;^75,76^ however, fewer studies have investigated its relationship with cognitive functions in late-adulthood individuals without dementia. One study reported a significant inverse association with cross-sectional working memory and executive functioning,^75^ and in the same cohort, a significant correlation was reported with cognitive functions at a 12-month follow-up.^76^

Despite the growing body of literature around blood-based biomarkers associated with age-related cognitive decline, few existing studies use detailed cognitive testing, which is required to identify early and potentially subtle differences in cognitive functioning. For instance, the use of the mini-mental state examination (MMSE) in many studies may lead to an underestimation of the relationship between cognitive function and plasma markers as the MMSE exhibits a ceiling effect in those without dementia^77^ and exhibits restricted ageing trends^78^ at odds with reports using psychometric psychological measurement.^79^ Associations between these plasma markers and longitudinal cognitive ageing differences require more detailed psychometric measurement to characterize differences more accurately among non-demented older adults. Further, much of the existing literature examines cross-sectional cognition and where longitudinal associations are examined, follow-up periods are often short. Longitudinal data are important to fully reflect the within-person dynamics of the cognitive ageing phenomenon. In the current study, we examine the association between cognitive decline and plasma p-tau181, Aβ42/40, NfL and GFAP in the Lothian birth cohort 1936 (LBC1936). This cohort offers the benefit of a narrow age range with a long follow-up period (∼10 years), which provides a focused insight into those who are exclusively in the transition phase from 70 to 80 years old, when the risk of cognitive decline starts to accelerate markedly.^80^

Research around plasma p-tau181 as a biomarker is burgeoning; however, relatively few studies have examined this phopsho-site in brain tissue. Synapse loss is the strongest pathological correlate of cognitive decline in Alzheimer’s disease, and region-specific synapse changes are also thought to contribute to age-related cognitive decline.^81^ Emerging evidence suggests that abnormal tau accumulation in synapses contributes to synapse degeneration.^82^ Previous work has reported evidence of p-tau accumulation in both AD and age-matched control brain tissue, specifically p-tau S202/Thr205 in crude homogenates^6^ and S396/404 and S202 in synaptoneurosome fractions.^83^ Further, astrocytic tau accumulation has also been reported in post-mortem AD cases,^84^ and p-tau181 has been reported in astrocytes in a number of tauopathies.^85^ The accumulation of p-tau181 in neurofibrillary tangles (NFTs) has been reported in AD post-mortem tissue, and the percentage of p-tau217 in NFTs has been shown to be increased in AD relative to aged controls.^86^ Furthermore, p-tau181, −205, −217 and −231 have been found to recognize less mature NFTs in AD, with p-tau181 potentially recognizing pre-tangles to a higher extent than more mature tangles.^87^ While there is evidence that p-tau181 is present in the brain in ageing without dementia,^86,88^ it is currently unclear whether it is present in synapses, astrocytes and NFTs in the ageing brain in the absence of dementia.

Levels of p-tau in blood increase with increasing brain Aβ accumulation,^89,90^ leading to the hypothesis that plaque-associated tau pathology may be the source of peripheral p-tau. Dystrophic neurites accumulate around plaques and usually contain pathological forms of tau.^91^ Membrane fragility has been reported in dystrophic neurites,^92^ which indicates that they may be a source of leaking of pathological tau into the parenchyma and ultimately the periphery. Dystrophic neurites also contain high levels of neurofilament proteins and are accompanied by astrogliosis,^91,93^ making them a potential source of peripheral GFAP and NfL.

In the current study, we examined whether plasma levels of p-tau181, Aβ42/40, NfL and GFAP are associated with cognitive decline in a large cohort of older adults without dementia, the LBC1936. We hypothesized that greater plasma p-tau181, NfL and GFAP, and lower levels of Aβ42/40 will be significantly associated with cognitive decline as measured by longitudinal changes in participant-suitable measure of cognitive function, the general cognitive factor (known as the *g* factor). A second aim of the current study is to examine whether p-tau181 is present in brain tissue of healthy ageing cases. We examined p-tau181 accumulation in synapses, astrocytes, NFTs and dystrophic neurites in post-mortem brain tissue and whether this is associated with pre-morbid cognitive decline.

## Materials and methods

### Participants

#### Plasma phosphorylated tau Lothian birth cohort 1936 sample

Plasma marker data and *APOE* genotypes were obtained from 200 participants in a longitudinal study of ageing, the LBC1936.^94-96^ The LBC1936 comprised participants of the Scottish Mental Survey 1947, who were attending school in Scotland in 1947 and were tested with a version of the Moray House Test No. 12.^97^ Between 2004 and 2007, these individuals were identified using the Community Health Index (lists of individuals registered with general practitioners) and media advertisements. In total, 1091 participants were recruited into the Lothian birth cohort 1936 aged just under 70 years old.^95^ To date, data have been collected at five waves, approximately every three years. Plasma samples were obtained at wave 2 (*M* age = 72.46, *SD* = 0.70) and wave 5 (*M* age = 82.03, *SD* = 0.46). Participants were not eligible for selection if there was evidence of neuroradiologically identified stroke. Participants were selected for inclusion if complete plasma data were available for both waves 2 and 5, and if cognitive data were available over waves 2, 3, 4 and 5. Selected participants were also opted-in for post-mortem tissue donation. Of the 200 participants selected, five participants were diagnosed with dementia by wave 5 follow-up and were removed from analyses. During a medical interview at each wave, participants provided a medical history, which included a self-reported diagnosis of diabetes, hypertension, high cholesterol and cardiovascular disease. Blood pressure was measured six times at each wave—three times sitting and three times standing—and the mean was taken for each. Blood pressure measurements were categorized as systolic pressure and diastolic pressure. High-density lipoprotein (HDL) cholesterol and low-density lipoprotein (LDL) cholesterol were measured in serum samples. Anxiety and depression were measured by the Hospital Anxiety and Depression Scale (HADS), a 14-item self-assessment questionnaire.^98^ Body mass index (BMI) was calculated for each participant (kg/m^2^). All participants provided informed consent according to the Declaration of Helsinki, and ethical approval was obtained from Multi-Centre Research Ethics Committee for Scotland (MREC/01/0/56; wave 1), the Lothian Research Ethics Committee (LREC/2003/2/29; wave 1) and the Scotland A Research Ethics Committee (07/MRE00/58; waves 2–5).

#### Post-mortem samples

Use of human tissue for post-mortem studies has been reviewed and approved by the Academic and Clinical Central Office for Research and Development medical research ethics committee (approval 15-HV-016) and the Edinburgh Brain Bank (research ethics committee approval 16/ES/0084). All donors provided informed consent (according to the Declaration of Helsinki) for tissue to be used for research purposes. Tissue from 33 donors was used in the current study (Table 1). BA20/21 was examined as this region is involved in higher-order cognitive functions such as learning and memory, and has been reported to exhibit a greater pathological burden of tau in the early stages of AD and cognitive decline.^99–101^ Donors were either healthy agers (HA), people who died in mid-life with no known neurological or psychiatric disorders (mid-life, ML), or those who died with both clinical and neuropathological diagnoses of AD. Healthy agers were enrolled in the LBC1936 study, however, only one HA participant was involved in both the post-mortem study and the plasma p-tau181 study. Data were available for donors in the HA group relating to pre-morbid lifetime cognitive decline computed from the Moray House Test Number 12 (MHT)^97^ at age 11 and at ages 70–76. MHT at age 70–76 was regressed onto MHT at age 11; participants with positive residuals were classified as lifetime cognitive resilient (LCR, *n* = 5 for biochemical studies, *n* = 8 for array tomography study and immunohistochemistry study) as their MHT at age 70–76 is above what would be expected based on age 11 MHT. Those with negative residuals were classified as experiencing lifetime cognitive decline (LCD, *n* = 6 for biochemical studies, *n* = 7 for array tomography study and *n* = 8 immunohistochemistry study). One HA participant involved in the array tomography sample was also a participant in the p-tau181 plasma sample.

**Table 1:** Post-mortem sample subject characteristics

BBN number	Group	Method	Age	PMI	Sex	Braak stage	Thal stage	Cognitive status
001.28406	HA	WB, AT, IHC	79	72	Male	2	2	LCD
001.32577	HA	WB, AT, IHC	81	74	Male	2	3	LCD
001.28793	HA	WB, AT, IHC	79	72	Female	2	1	N/A
001.28794	HA	WB, AT, IHC	79	61	Female	1	0	LCR
001.26495	HA	WB, AT, IHC	78	39	Male	1	1	LCD
001.28797	HA	WB, AT, IHC	79	57	Male	0	0	LCR
001.31495	HA	WB, AT,	81	38	Male	6	4	LCR
001.28402	HA	WB, AT, IHC	79	49	Male	1	2	LCD
001.19686	HA	WB, AT, IHC	77	75	Female	1	1	LCD
001.34131	HA	WB, AT, IHC	82	95	Male	4	3	LCD
001.29082	HA	WB, AT, IHC	79	80	Female	3	5	LCR
001.29086	HA	WB, AT, IHC	79	68	Female	0	1	LCR
001.35215	HA	AT, IHC	82	40	Male	1	0	LCR
001.35549	HA	AT, IHC	82	56	Male	1	1	LCR
001.35866	HA	AT, IHC	83	78	Female	4	4	LCD
001.36135	HA	AT, IHC	84	30	Female	2	1	LCR
001.36435	HA	IHC	84	95	Male	2	1	LCR
001.36809	HA	IHC	85	3	Male	2	1	LCD
001.24479	ML	WB	46	76	Female	0	0	N/A
001.29906	ML	WB	51	52	Male	N/A	N/A	N/A
001.33613	ML	WB	46	99	Female	0	0	N/A
001.30169	ML	WB	48	58	Male	N/A	N/A	N/A
001.34244	ML	WB	49	69	Female	0	0	N/A
001.29466	ML	WB	39	76	Male	0	0	N/A
001.24342	ML	WB	33	47	Male	0	0	N/A
001.28792	ML	WB	58	51	Male	N/A	N/A	N/A
001.30972	ML	WB	34	99	Male	0	0	N/A
001.26976	ML	WB	19	101	Male	0	0	N/A
001.36066	AD	WB	94	29	Male	6	5	N/A
001.29695	AD	WB, AT, IHC	86	72	Male	6	5	N/A
001.35183	AD	WB	74	75	Male	6	5	N/A
001.30142	AD	WB, AT, IHC	88	112	Female	6	5	N/A
001.29135	AD	WB, IHC	90	73	Male	6	3	N/A
001.35564	AD	WB	90	52	Female	6	5	N/A
001.30883	AD	WB, AT	61	69	Female	6	5	N/A
001.29521	AD	WB, IHC	95	96	Male	6	5	N/A
001.30973	AD	WB	89	96	Female	6	5	N/A
001.36328	AD	WB	71	81	Male	6	5	N/A
001.36346	AD	WB	90	60	Female	6	5	N/A
001.32929	AD	AT	85	80	Female	6	5	N/A
001.35535	AD	AT	83	95	Female	6	4	N/A
001.35811	AD	AT	83	72	Male	6	4	N/A
001.26718	AD	AT	78	74	Male	6	5	N/A
001.33636	AD	IHC	93	43	Male	6	5	N/A
001.31499	AD	IHC	85	78	Male	6	5	N/A
001.28771	AD	IHC	85	91	Male	6	5	N/A
001.28410	AD	IHC	62	109	Female	6	5	N/A
001.24526	AD	IHC	79	65	Male	6	0	N/A

BBN = brain bank number; AD = Alzheimer’s disease; AT = array tomography; WB = western blot; IHC = immunohistochemistry; *APOE* = apolipoprotein E; HA = healthy ager; ML = mid-life; LCD = lifetime cognitive decline; LCR = lifetime cognitive reserve; PMI = post-mortem interval (hours).

### Plasma assays

Plasma assays were conducted at the biomarker lab at the UK Dementia Research Institute at UCL. Plasma p-tau181 (*i.e.* tau phosphorylated at threonine 181) concentrations were measured by the Quanterix single molecule array (SIMOA) assay, and the Quanterix SIMOA Human Neurology 4-Plex E (NP4E) assay was used to measure GFAP, NfL Aβ40 and Aβ42 concentrations according to manufacturer’s instructions. Briefly, samples were thawed at 21°C, and centrifuged at 10 000 RCF for 5 minutes at 21°C. Calibrators (neat) and samples (plasma: 1:4 dilution) were measured in duplicates. All samples were analysed using the same batch of reagents. A four-parameter logistic curve fit data reduction method was used to generate a calibration curve. Two control samples of known concentration of the protein of interest (high-ctrl and low-ctrl) were included as quality control. Mean coefficient of variation (CV) was 8.29% for plasma p-tau181, and intra-assay and inter-assay CVs were less than 17% and 10%, respectively, as determined by 10 quality controls for the remaining markers.

### Computation of change in general cognitive function

Participants in the LBC1936 study were given a detailed battery of neuropsychological tests at waves 2 (age ∼73), 3 (age ∼76), 4 (age ∼79) and 5 (age ∼82). In the current study, we used the following six subtests from the Wechsler Adult Intelligence Scale-III UK^102^ (WAIS-III) to compute a slope representing general fluid cognitive (*g*) decline (from here referred to as *g* factor change): matrix reasoning, letter-number sequencing, block design, symbol search, digit symbol coding and digit span backwards. *g* factor was derived from the factor-of-curves model. This general cognitive factor was chosen as it accounts for much of the variation in change in the specific cognitive domains; there are high correlations among the age-related changes between the various cognitive domains.^103^ Looking at contributions to cognitive change, most predictors are associated with the change in the general cognitive factor rather than to the specific domains (after adjusting for change that is common across all cognitive domains). Using this general cognitive factor change has the following advantages: it is the locus of most of the reliable age-related change; it is the destination for most of the ‘prediction’ of age-related change, and, by using this one informative variable, we can limit the type 1 error rate. Individual scores of cognitive ageing were computed for the total LBC1936 sample by fitting a Factor of Curves model in a structural equation-modelling framework using full information maximum likelihood estimation in *R*, using the *lavaan* package.^103^ The latent slope scores were extracted for further analysis.

## Polygenic risk score for Alzheimer’s disease

A polygenic risk score (PRS) for AD was created in LBC1936 using PRSice-2.^104^ Summary data from the largest clinically ascertained AD genome-wide association study, which included 21 982 people with AD and 41 944 controls, was used.^105^ As recommended by Leonenko *et al.*,^106^ a 2.2-Mb region of the genome containing the *APOE* region was removed prior to creating the PRS, and a *P*-value threshold of 0.1 was used for single nucleotide polymorphism (SNP) selection. SNPs with a minor allele frequency <0.01 were removed, and clumping was used to obtain SNPs in linkage equilibrium with an *r*2 < 0.10 within a 250-bp window.

### Immunoblotting

In the post-mortem study, total brain homogenates and synaptoneurosomes (SN) were prepared according to Tai *et al.*^83^ Freshly frozen human brain tissue (BA20/21) of 200 mg was homogenized in 1-mL buffer (25-mM/L HEPES pH 7.5, 120-mM/L NaC1, 5-mM/L KCL 1-mM/L MgC1_2_, 2-mM/L CaC1_2_), with protease inhibitors (Roche complete mini) and phosphatase inhibitors (Millipore, Watford, UK). The homogenate was passed through an 80-μm nylon filter (Millipore, Watford, UK), and a 300-μL aliquot was saved and mixed with buffer (100-mM/L Tris-HC1 pH 7.6, 4% SDS, protease inhibitor cocktail EDTA-free 100 × Thermo Fisher Scientific, Loughborough, UK) to prepare the crude homogenate. The remainder of the homogenate was passed through a 5-μm filter (Millipore, Watford, UK) then centrifuged at 1000×g for 5 minutes. The supernatant was discarded, and the pellet was washed with buffer and centrifuged again, yielding the synaptoneurosome pellet. Protein concentrations were determined using a protein assay (Thermo Fisher Scientific, Loughborough, UK). Protein per sample of 20 μg was electrophoresed in 4–12% Bis-Tris polyacrylamide gels (Invitrogen, Paisley, UK). Proteins were electro-transferred to nitrocellulose membranes (Thermo Fisher Scientific, Loughborough, UK) using the iBlot^™^ Dry Blotting system (#IB21001, Invitrogen, Paisley, UK). Revert 700 Total Protein Stain was used to quantify total protein (Li-Cor, Cambridge, UK). Membranes were incubated in block buffer, then incubated in primary antibodies in blocking buffer [p-tau181 (1:500, Invitrogen AT270 #MN1050) and total tau (1:500, Tau13 BioLegend #MMS-520R)]. Membranes were washed and incubated with secondary antibodies (1:5000, Li-Cor Biosciences), rinsed and imaged using the Odyssey Imaging system, and analysed using Open Image Studio Lite. Example un-cropped blots are shown in Supplementary Fig. 1.

## Array tomography

Samples from BA20/21 were trimmed into blocks and fixed in 4% paraformaldehyde for 3 hours.^107^ Samples were dehydrated, embedded in LR White resin and cut into ribbons of 70-nm serial sections. Ribbons were outlined with a hydrophobic pen, rehydrated with 50-mM glycine in TBS, washed in TBS and incubated in blocking buffer (0.05% Tween, 0.1% fish skin gelatine in TBS) for 45 minutes. Primary antibody solution of 150 uL (p-tau181 (1:50, Invitrogen #MN1050), GFAP (1:100, Abcam #AB4674), diluted in block buffer was applied and incubated overnight at 4°C. Ribbons were washed with TBS and 150 uL of secondary antibody solution diluted in block buffer was incubated for 45 minutes at room temperature [goat anti-chicken IgY H&L Alexa Fluor 405 (1:100, Abcam #A48260) and donkey anti-mouse IgG H&L Alexa Fluor 647 (1:50, Abcam #A31571)]. Ribbons were washed with TBS and 150 uL of Alexa Fluor 488 labelled anti-synaptophysin antibody solution (1:200, Abcam #196379) diluted in block solution was then applied for 1 hour. Ribbons were washed with TBS, then distilled H_2_O. They were mounted on glass slides using Immu-Mount. Images were acquired on a AxioImager Z2 with 63 × 1.4 numerical aperture oil objective and analysed using FIJI, MATLAB (version 2018a) and Docker to run python-based analysis scripts, which are freely available on GitHub https://github.com/arraytomographyusers/Array_tomography_analysis_tool.

### Immunofluorescence staining and quantification of pathology

Parrafin sections of formalin-fixed tissue from BA20/21 were de-waxed and stained with primary antibodies to p-tau181 (R&D Systems MN1050, mouse IgG) and GFAP (Agilent Z0334, rabbit polyclona). Secondary antibodies used were donkey anti-rabbit labelled with AlexaFluor 594 (Abcam A21207) and donkey anti-mouse labelled with AlexaFluor 647 (Abcam A31571). After rinsing secondaries, sections were incubated in 0.01% Thioflavine S in 50% ethanol to label amyloid fibrils (plaques and neurofibrillary tangles). Ten images per slide were acquired on Zeiss AxioImager microscopes with a 20 × air objective. Regions of interest were selected with plaque or tangle pathology. Images were analysed by a blind observer in Image J. Plaques were classified as p-tau181 dystrophy-positive if there were at least two swellings of greater than 3-μm diameter in contact with ThioS-positive plaques. Neurofibrillary tangles were identified by flame or oval shaped ThioS staining and absence of GFAP staining to exclude astrocytic tau pathology. Tangles were classified as p-tau181-positive or negative. See Supplementary Fig. 2 for example classifications.

## Statistical analysis

R Studio (version 4.0.4) was used for all data analysis. Data were tested for normality by inspection of histograms and Shapiro–Wilk tests. Group differences were analysed using *t*-tests, Wilcoxon rank-sum and Kruskal–Wallis where appropriate. Correlations were conducted using Pearson’s *r* or Spearman’s rho dependent on normality of data. Cohen’s *d* was used as a measure of effect size.^108^ To examine whether sex, age and *APOE* status were associated with change in plasma biomarkers, linear regression models were fitted where change in plasma marker was regressed onto sex, age and *APOE* individually. To examine whether baseline plasma p-tau181, NfL, GFAP and Aβ42/40 could predict change in cognitive function, two models were fitted. The first where *g* factor change was regressed onto sex, baseline age (years) and baseline marker of interest. The second model added education (years), age 11 IQ and *APOE* status (categorical variable of *APOE4* carrier or *APOE4* non-carrier). Baseline marker values were log-transformed to adjust for a non-normal distribution. Change in plasma markers was computed by regressing values at wave 5 onto values at wave 2. The standardized residuals from this model were then entered into identical models as above. Linear model fit was assessed by inspection of residuals, and all linear regression assumptions were met. Analyses were repeated for the sample stratified by sex to examine any sex differences. Identical models were fitted (with the exception of the sex variable being removed) for males and females separately. Sensitivity analyses were conducted to examine whether associations remained robust to the inclusion of medical comorbidities and a PRS for AD. Standardized coefficients are reported (β) with bootstrapped 95% confidence intervals (CI) from 1000 replications. Significance was reported when *P* < 0.05. Statistical tests were two-tailed.

Kruskal–Wallis tests were used to examine group differences in brain p-tau181 in total homogenate and synaptoneurosome preparations, and Wilcoxon rank-sum tests were used to examine differences between HA with age-related cognitive decline and those with maintained cognition. Array tomography data were analysed using linear mixed effects models, with cohort, sex, age and *APOE* genotype entered as fixed-effects, and case number as a random effect to avoid pseudo-replication. ANOVAs were run on linear mixed effects models with Satterthwaite’s method of estimation of degrees of freedom. We used linear regressions to examine the association between cohort (AD or HA) and the percentage of plaques containing p-tau181 dystrophies, and the percentage of NFTs positive for p-tau181, respectively. Models included cohort, sex and *APOE* status. *APOE* status was entered as a binary variable (*APOE4* carrier versus non-carrier). Outcome variables were log-transformed where appropriate, to meet model assumptions. *Post-hoc* comparisons were performed with Tukey-corrected estimated marginal means.

### Data availability

Data collected *in vivo* can be requested from the Lothian birth cohorts research group at the University of Edinburgh (ed.ac.uk/lothian-birth-cohorts/data-access-collaboration). Anonymized post-mortem data are available on Edinburgh datashare (https://doi.org/10.7488/ds/3826).

## Results

### Plasma markers and cognitive decline in Lothian birth cohort 1936

#### Analysis of selected sample versus total Lothian birth cohort 1936

Cognitive and plasma-based data were available for two time-points when participants were a mean age of 72.46 (*SD* = 0.70) and 82.02 years (*SD* = 0.46). Demographic information is provided in Table 2. The LBC1936 sample contains a total of 1091 participants, with 195 selected for inclusion for the current study. We examined group differences between those included in the current study and those in the total LBC1936 who were not. There were no significant differences in age at wave 2 (*t* = 1.83, *P* = 0.07) or wave 5 (*t* = 1.37, *P* = 0.17), nor in proportion of males and females (χ^2^ = 0.38, *P* = 0.54). Those included in the current study had significantly higher age 11 IQ than those not included (*W* = 63927, *P* < 0.01, *d* = 0.30, 95% CI = 0.14–0.46), as well as significantly more years of formal education (*W* = 76828, *P* = 0.01, *d* = 0.26, 95% CI = 0.11–0.41). Further, there was a significant difference in *g* factor decline (*W* = 65029, *P* < 0.01, *d* = 0.41, 95% CI = 0.25–0.56) where those included experienced less cognitive decline than those not included.

**Table 2:** Plasma marker study LBC1936 subject characteristics

Variable	Wave 2	Wave 5
Sex (F:M, *N*)	102:93	101:91
Age (years, mean ± SD)	72.46 ± 0.70	82.02 ± 0.46
*APOE* ε4 carriers, (*N* (%))	52 (26.67%)	51 (26.56%)
Plasma p-tau181 (pg/mL, mean ± SD)	24.74 (13.35)	30.34 (18.62)
Plasma GFAP (pg/mL, mean ± SD)	94.57 ± 37.86	133.55 ± 55.76
Plasma NfL (pg/mL, mean ± SD)	14.85 ± 4.54	21.53 ± 8.55
Plasma Aβ1–40 (pg/mL, mean ± SD)	50.24 ± 14.80	75.99 ± 17.06
Plasma Aβ1–42 (pg/mL, mean ± SD)	3.99 ± 1.42	6.10 ± 1.59
Plasma Aβ1–40/1–42 ratio (pg/mL, mean ± SD)	0.08 ± 0.02	0.08 ± 0.02
Age 11 IQ (mean ± SD)	103.83 (14.04)	
Education (Years, mean ± SD)	11.01 (1.16)	

*APOE* = apolipoprotein E; p-tau181 = phosphorylated tau 181; GFAP = glial fibrillary acidic protein; NfL = neurofilament-light; Aβ = amyloid-beta; IQ = intelligence quotient.

We examined group differences in medical comorbidities and vitals between those selected for inclusion in the current study and those in the total LBC1936 who were not. A smaller proportion of participants included in the current study had a diagnosis of diabetes (χ^2^ = 4.74, *P* = 0.03) and cardiovascular disease (χ^2^ = 4.74, *P* = 0.03). Furthermore, compared to the rest of the sample, those included in the current study had significantly elevated LDL cholesterol (*t* = −2.08, *P* = 0.04), lower HADS anxiety scores (*W* = 78548, *P* < 0.01) and lower HADS depression scores (*W* = 76765, *P* < 0.01). There were no significant group differences in SBP, DBP, HDL cholesterol or BMI.

#### Descriptive statistics

**Figure 1:**
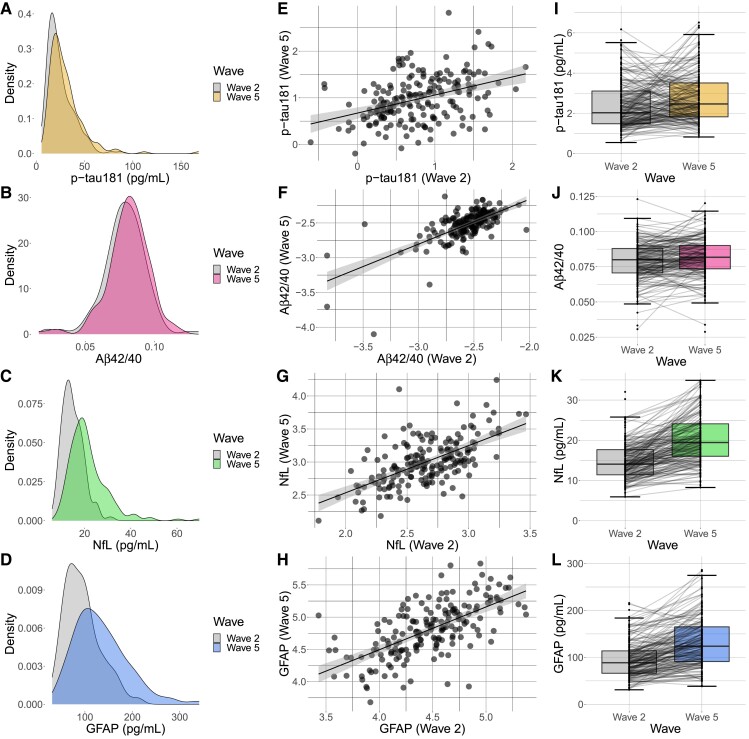
**Plots showing change in plasma markers between wave 2 and wave 5.** (**A**–**D**) Density plots of plasma markers at wave 2 and wave 5. (**E**–**H**) Scatterplot of log-transformed plasma markers at wave 2 and 5 with regression line and shaded 95% confidence intervals. (**I**–**L**) Boxplots of plasma markers at wave 2 and wave 5, central line delineates the median, lines connect each participant’s biomarker levels between wave 2 and wave 5. Each data-point represents one participant (*n* = 192).

Descriptive plots of plasma markers and marker change are shown in Fig. 1. Between wave 2 and wave 5 (follow-up ∼10 years), there were significant elevations in p-tau181 levels (*W* = 12867, *P* < 0.01, *d* = 0.30, 95% CI = 0.15–0.44), GFAP (*W* = 10049, *P* < 0.01, *d* = 0.88, 95% CI = 0.71–1.05), NfL (*W* = 7946, *P* < 0.01, *d* = 0.94, 95% CI = 0.76–1.11), Aβ40 (*W* = 4224, *P* < 0.01, *d* = 1.38, 95% CI = 1.18–1.59), Aβ42 (*W* = 5452, *P* < 0.01, *d* = 1.37, 95% CI = 1.17–1.57) and Aβ42/40 (*W* = 15537, *P* = 0.04, *d* = 0.24, 95% CI = 0.09–0.39).

Sex was significantly associated with 10-year change in Aβ42/40 ratio (β = −0.18, 95% CI = −0.69 to −0.13, *P* = 0.01), with a greater decrease in Aβ42/40 in females relative to males. Sex was not associated with change in p-tau181 (β = −0.01, 95% CI = −0.27–0.30, *P* = 0.94), NfL (β = 0.05, 95% CI = −0.19–0.40, *P* = 0.54) and GFAP (β = 0.11, −0.08–0.50, *P* = 0.15). *APOE* status was not associated with change in any plasma biomarker (p-tau: β = 0.10, 95% CI = −0.11–0.53, *P* = 0.17; NfL: β = −0.04, 95% CI = −0.40–0.22, *P* = 0.62; GFAP: β = 0.02, 95% CI = −0.28–0.38, *P* = 0.84; Aβ42/40: β = 0.10, 95% CI = −0.06–0.54, *P* = 0.19). Age at baseline significantly predicted greater increases in NfL (β = 0.18, 95% CI = 0.09–0.43, *P* = 0.01) and GFAP (β = 0.23, 95% CI = 0.12–0.51, *P* < 0.01). Age was not associated with change in p-tau181 (β = −0.05, 95% CI = −0.27–0.13, *P* = 0.50) or Aβ42/40 (β = −0.11, 95% CI = −0.41–0.06, *P* = 0.13).

### Plasma phosphorylated tau 181, glial fibrillary acidic protein and neurofilament-light predict cognitive decline

**Figure 2:**
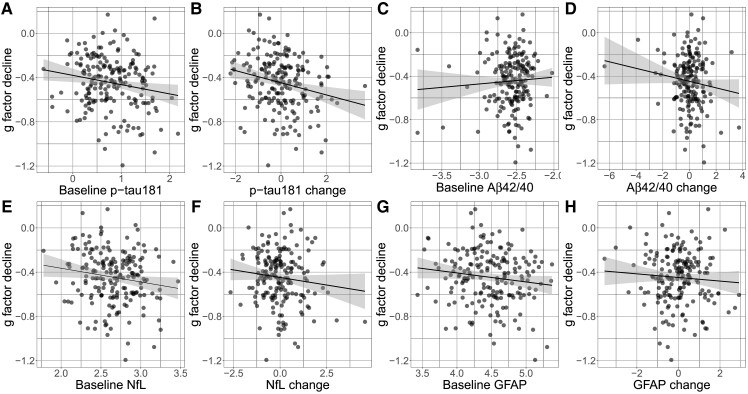
**Scatterplots of *g* factor decline and (log transformed) plasma markers. Linear regressions were performed for each marker.** (**A**) baseline plasma p-tau181 (*β* = −0.17, *P* = 0.02, *n* = 195), (**B**) plasma p-tau181 change (*β* = −0.23, 95%, *P* = <0.01, *n* = 192), (**C**) baseline Aβ42/40 (*β* = 0.05, *P* = 0.47, *n* = 195), (**D**) Aβ42/40 change (*β* = 0.05, *P* = 0.47, *n* = 192), (**E**) baseline NfL (*β* = −0.16, *P* = 0.03, *n* = 195), (**F**) NfL change (*β* = −0.14, *P* = 0.07, *n* = 192), (**G**) baseline GFAP (*β* = −0.16, *P* = 0.02, *n* = 195), (**H**) GFAP change (*β* = −0.10, *P* = 0.17, *n* = 192). Each data-point represents one participant.

Regression coefficients are provided in Table 3. In model one (baseline p-tau181, age and sex), p-tau181 at age 73 significantly predicted subsequent 10-year *g* factor decline (β = −0.17, 95% CI = −0.15 to −0.01, *P* = 0.02), where a 1 SD higher baseline level of p-tau181 is associated with 0.17 SD decrease in *g* factor (Fig. 2A). This association was robust to the inclusion of age 11 IQ, education years and *APOE* status (Model 2; β = −0.17, 95% CI = −0.16 to −0.01, *P* = 0.03). Next, *g* factor change was regressed onto p-tau181 change, sex and age. P-tau181 change was a significant predictor of *g* factor decline (β = −0.23, 95% CI = −0.09 to −0.02, *P* = <0.01). When including covariates age 11 IQ, education years and *APOE* status, p-tau181 change remained a significant predictor of *g* factor decline (β = −0.21, 95% CI = −0.08 to −0.01, *P* = < 0.01), where a 1 SD increase in p-tau181 change was associated with a 0.21 SD decrease in *g* factor (see Fig. 2B).

**Table 3:** Association of plasma markers with *g* factor decline

	Model 1						Model 2					
	Females		Males		Total sample		Females		Males		Total sample	
Wave 2	*β* (95% CI)	*P*	*β* (95% CI)	*P*	*β* (95% CI)	*P*	*β* (95% CI)	*P*	*β* (95% CI)	*P*	*β* (95% CI)	*P*
p-tau181	−0.27 (−0.12 to −0.02)	<0.01*	−0.10 (−0.05–0.01)	0.35	−0.17 (−0.15 to −0.01)	0.02*	−0.24 (−0.26 to −0.03)	0.03*	−0.20 (−0.13–0.07)	0.07	−0.17 (−0.16 to −0.01)	0.03*
NfL	−0.15 (−0.02–0.01)	0.14	−0.17 (−0.02–0.01)	0.12	−0.16 (−0.24 to −0.01)	0.03*	−0.18 (−0.29–0.03)	0.11	−0.17 (−0.36–0.05)	0.12	−0.17 (−0.25 to −0.01)	0.03*
GFAP	−0.12 (−0.01–0.01)	0.23	−0.21 (−0.01–0.01)	0.06	−0.16 (−0.19 to −0.01)	0.02*	−0.14 (−0.24–0.03)	0.23	−0.19 (−0.22–0.02)	0.09	−0.16 (−0.19 to −0.01)	0.04*
Aβ1-40	0.16 (−0.01–0.01)	0.12	−0.12 (−0.01–0.01)	0.28	0.03 (−0.01–0.01)	0.71	0.16 (−0.03–0.27)	0.18	−0.11 (−0.24–0.09)	0.34	0.02 (−0.01–0.01)	0.83
Aβ1-42	0.15 (−0.01–0.05)	0.14	−0.05 (−0.05–0.03)	0.67	0.04 (−0.02–0.03)	0.54	0.17 (−0.05–0.22)	0.15	−0.05 (−0.17–0.13)	0.69	0.05 (−0.02–0.04)	0.55
Aβ42/40	0.01 (−0.19–0.27)	0.94	0.10 (−0.20–0.30)	0.34	0.05 (−0.01–0.01)	0.47	0.03 (−0.17–0.30)	0.83	0.10 (−0.25–0.36)	0.40	0.07 (−0.01–0.01)	0.40

Change between Wave 2 and Wave 5	Females		Males		Total sample		Females		Males		Total sample	
	*β* (95% CI)	*P*	*β* (95% CI)	*P*	*β* (95% CI)	*P*	*β* (95% CI)	*P*	*β* (95% CI)	*P*	*β* (95% CI)	*P*
p-tau181	−0.26 (−0.11 to −0.02)	0.01*	−0.22 (−0.09 to −0.01)	0.03*	−0.23 (−0.09 to −0.02)	< 0.01*	−0.24 (−0.13–0.02)	0.03*	−0.20 (−0.09–0.01)	0.07	−0.21 (−0.08 to −0.01)	< 0.01*
NfL	−0.27 (−0.10–0.01)	0.01*	−0.01 (−0.05–0.05)	0.90	−0.14 (−0.06 to −0.03)	0.07	−0.29 (−0.11–0.01)	0.01*	0.02 (−0.05–0.06)	0.85	−0.11 (−0.07 to −0.02)	0.16
GFAP	−0.19 (−0.08–0.01)	0.07	−0.01 (−0.06–0.06)	0.90	−0.10 (−0.05–0.02)	0.17	−0.22 (−0.08–0.04)	0.05	−0.01 (−0.06–0.07)	0.99	−0.11 (−0.06 to −0.02)	0.15
Aβ1–40	−0.05 (−0.06–0.03)	0.67	0.12 (−0.02–0.09)	0.29	0.03 (−0.01–0.05)	0.66	−0.06 (−0.12 to −0.01)	0.63	0.11 (−0.02–0.09)	0.35	0.02 (−0.02–0.04)	0.76
Aβ1–42	−0.18 (−0.09–0.01)	0.08	0.17 (−0.01–0.12)	0.12	−0.02 (−0.04–0.04)	0.82	−0.18 (−0.10–0.03)	0.12	0.16 (−0.02–0.11)	0.15	−0.01 (−0.04–0.03)	0.89
Aβ40/42	−0.15 (−0.07–0.01)	0.15	−0.01 (−0.08–0.08)	0.92	−0.09 (−0.06 to −0.01)	0.22	−0.14 (−0.09–0.01)	0.23	0.01 (−0.08–0.08)	0.99	−0.08 (−0.07–0.01)	0.34

Model 1: adjusted for age, sex; Model 2: adjusted for age, sex, *APOE* status, years of education, age 11 IQ.

*P < 0.05.

In model one (baseline Aβ42/40, age and sex), Aβ42/40 did not significantly predict *g* factor decline (β = 0.05, 95% CI = −0.01–0.01, *P* = 0.47; Fig. 2C). This remained non-significant when education years, age 11 IQ and *APOE* status were included in the model (β = 0.07, 95% CI = −0.01–0.01, *P* = 0.40). Change in Aβ42/40 also did not significantly predict *g* factor decline in either model one (β = 0.05, 95% CI = −0.01–0.01, *P* = 0.47) or model two (β = 0.07, 95% CI = −0.01–0.01, *P* = 0.40).

In model one (baseline NfL, age and sex), NfL levels significantly predicted *g* factor decline (β = −0.16, 95% CI = −0.24 to −0.01, *P* = 0.03) where a 1 SD higher baseline NfL is associated with a 0.16 SD decrease in *g* factor (see Fig. 2E). Plasma NfL remained a significant predictor when adding education years, age 11 IQ and *APOE* status to the model (β = −0.17, 95% CI = −0.25 to −0.01, *P* = 0.03). Conversely, NfL change between wave 2 and wave 5 did not significantly predict *g* factor decline in either model one (β = −0.14, 95% CI = −0.06 to −0.03, *P* = 0.07) or model two (β = −0.11, 95% CI = −0.07 to −0.02, *P* = 0.16; Fig. 2F).

In model one (baseline GFAP, age and sex), GFAP significantly predicted *g* factor decline (β = −0.16, 95% CI = −0.19 to −0.01, *P* = 0.02) where a 1 SD higher baseline GFAP level is associated with a 0.16 SD decrease in *g* factor (Fig. 2G). When education years, age 11 IQ and *APOE* status were included in the model, GFAP remained a significant predictor of *g* factor decline (β = −0.16, 95% CI = −0.19 to −0.01, *P* = 0.04). GFAP change was not a significant predictor of *g* factor change in either model one (β = −0.10, 95% CI = −0.05 to −0.02, *P* = 0.17) or model two (β = −0.11, 95% CI = −0.06 to −0.02, *P* = 0.15; Fig. 2H).

#### Sex-stratified analyses

Regression coefficients are provided in Table 3. In model one (baseline p-tau181 and age), p-tau181 significantly predicted *g* factor decline in females only (*β* = −0.27, 95% CI = −0.12 to −0.02, *P* < 0.01), where a 1 SD higher baseline p-tau181 was associated with a 0.27 SD decrease in *g* factor. In model two (age, education, age 11 IQ and *APOE* status), this association remained significant (*β* = −0.24, 95% CI = −0.26 to −0.03, *P* = 0.03). In males, baseline p-tau181 was not associated with *g* factor decline in either model one (*β* = −0.10, 95% CI = −0.05–0.01, *P* = 0.35) or two (*β* = −0.20, 95% CI = −0.13–0.07, *P* = 0.07). Next, *g* factor change was regressed onto p-tau181 change and age. p-tau181 change was a significant predictor of *g* factor decline in both females (β = −0.26, 95% CI = −0.11 to −0.02, *P* = 0.01) and males (β = −0.23, 95% CI = −0.09 to −0.02, *P* < 0.01). When including covariates age 11 IQ, education years and *APOE* status, p-tau181 change remained a significant predictor of *g* factor decline (β = −0.21, 95% CI = −0.08 to −0.01, *P* = < 0.01), where a 1 SD increase in p-tau181 change was associated with a 0.21 SD decrease in *g* factor (see Fig. 2B).

#### Sensitivity analyses

##### Medical comorbidities

We examined the association between several medical comorbidities and each plasma biomarker in linear regression models adjusted for age and sex. Blood pressure was a significant predictor of baseline p-tau181 (diastolic: *β* = 0.14, 95% CI = 0.01–0.05, *P* = 0.04; systolic: *β* = 0.17, 95% CI = 0.01–0.03, *P* = 0.01), where higher blood pressure is associated with greater p-tau181 values. HDL cholesterol was a significant predictor of Aβ1–40 (*β* = −0.18, 95% CI = −13.58 to −2.24, *P* = 0.02) and Aβ1–42 (*β* = −0.16, 95% CI = −1.04 to −0.04, *P* = 0.04), with greater HDL levels predicting lower Aβ values. Body mass index was a significant predictor of plasma NfL (*β* = −0.17, 95% CI = −0.36 to −0.05, *P* = 0.02), where a 1 SD higher BMI was associated with a 0.17 SD decrease in NfL. There were no other significant associations between comorbidities and plasma biomarkers (full results shown in Supplementary Table 1).

Next, linear regressions were repeated to examine whether significant comorbidities mediate the association between the relevant plasma marker and cognitive function. In model one (baseline p-tau181, age, sex, SBP and DBP), p-tau181 remained a significant predictor of cognitive function (*β* = −0.16, 95% CI = −0.15 to −0.01, *P* = 0.04). When including covariates age 11 IQ, education years and *APOE* status, baseline p-tau181 did not significantly predict cognitive function (*β* = −0.15, 95% CI = −0.15 to −0.01, *P* = 0.06). When SBP and DBP were added to model 1, p-tau181 change remained a significant predictor of *g* factor decline (*β* = −0.24, 95% CI = −0.09 to −0.02, *P* < 0.01). This association remained robust to the addition of age 11 IQ, education years and *APOE* status (*β* = −0.22, 95% CI = −0.08 to −0.02, *P* < 0.01). Next, BMI was included in models examining the association between NfL and *g* factor decline. In model 1 (baseline NfL, age, sex and BMI), NfL remained a significant predictor of *g* factor decline (β = −0.17, 95% CI = −0.26 to −0.03, *P* = 0.02). This association remained robust in model 2 (β = −0.18, 95% CI = −0.28 to −0.03, *P* = 0.02). Next, HDL cholesterol was included in models examining Aβ42/40 and *g* factor decline. In model one (baseline Aβ42/40, age, sex and HDL cholesterol), Aβ42/40 remained a non-significant predictor of *g* factor decline (β = 0.06, 95% CI = −0.01–0.23, *P* = 0.40). This remained non-significant when education years, age 11 IQ and *APOE* status were included in the model (β = 0.07, 95% CI = −0.09–0.25, *P* = 0.34).

##### Alzheimer’s disease polygenic risk score

We examined the association between each plasma biomarker and *g* factor decline when including a PRS for Ad, which does not include the *APOE* region of the genome. In the total sample, baseline p-tau181, NfL, GFAP and Aβ42/40 were not significant predictors of *g* factor decline when the AD PRS was included in Models 1 and 2 (results shown in Supplementary Table 2). In females only, baseline p-tau181 remained a significant predictor of *g* factor decline (β = −0.22, 95% CI = −0.08 to −0.01, *P* = 0.04), although this did not remain significant when age 11 IQ, years of education and *APOE* status were included (β = −0.23, 95% CI = −0.09 to −0.01, *P* = 0.05).

Next, the association between change in plasma biomarkers and *g* factor decline was examined when adjusting for AD PRS. Change in plasma p-tau181 remained a significant predictor of *g* factor decline in the total sample (β = −0.19, 95% CI = −0.07 to −0.02, *P* < 0.01), which remained significant when including age 11 IQ, years of education and *APOE* status (β = −0.18, 95% CI = −0.07 to −0.01, *P* = 0.03). However, there were no significant associations in males and female separately (see Supplementary Table 2). In the total sample, there were no significant associations between *g* factor decline and change in NfL, GFAP or Aβ42/40. However, in females, change in NfL over ∼10 years predicted *g* factor decline over the same period (β = −0.24, 95% CI = −0.09–0.01, *P* = 0.02). This association was robust to the addition of years of education, age 11 IQ and *APOE* status (β = −0.26, 95% CI = −0.11–0.01, *P* = 0.03).

### Post-mortem analyses show plasma phosphorylated tau 181 in synapses, neurofibrillary tangles and dystrophic neurites

Thirty-three cases were used for western blot analyses of brain tissue, with 12 HA, 10 mid-life and 11 Alzheimer’s cases. Individual case details are provided in Table 1. Between the three groups, there were no significant differences in sex (χ^2^, *df* = 2, *P* = 0.75) or in PMI (*H* = 0.61, *df* = 2, *P* = 0.73). Both AD and HA donors were significantly older than mid-life donors (*H* = 41.91, *df* = 2, *P < 0.01)*, although there was no significant difference between Alzheimer’s and HA donors (*P* = 0.06).

**Figure 3:**
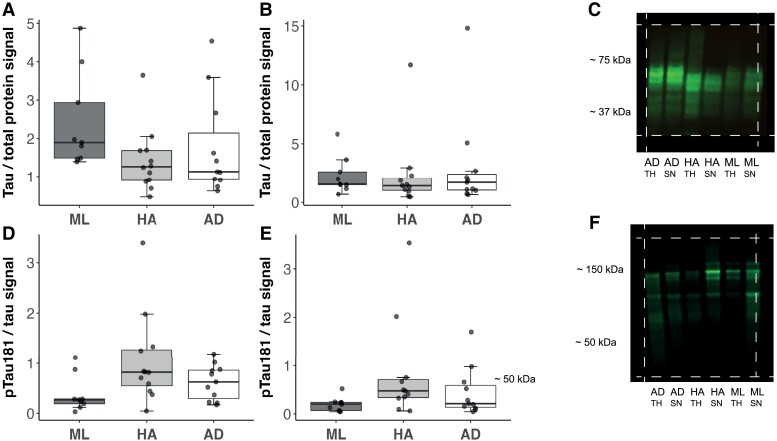
**Boxplots of total tau and phosphorylated tau across Alzheimer’s cases, mid-life controls and healthy agers.** Kruskal–Wallis tests were used to test group differences in (**A**) total tau in total homogenates (*H* = 6.12, *df* = 2, *P* = 0.05), (**B**) total tau in synaptoneurosomes (*H* = 0.83, *df* = 2, *P* = 0.66), (**C**) representative western blot of total tau, (**D**) p-tau181 in total homogenate (*H* = 5.59, *df* = 2, *P* = 0.06), (**E**) p-tau181 in synaptoneurosomes (*H* = 5.38, *df* = 2, *P* = 0.07) and (**F**) representative western blot of p-tau181. Each data-point in boxplots represents one participant. Total tau and p-tau181 were stained on separate gels with identical samples and gels/blots were processed in parallel. Cropped gel presented to aid understanding of data. White dashed lines indicate where image has been cropped. Uncropped images can be found in Supplementary Fig. 1. AD = Alzheimer’s disease (*n* = 11); ML = mid-life (*n* = 10); HA = healthy agers (*n* = 12); TH = total homogenate; SN = synaptoneurosome.

As seen in Fig. 3A, across the three groups, there were no significant differences in total tau levels (*H* = 6.12, *df* = 2, *P* = 0.05), in total homogenate nor in synaptoneurosome preparations (*H* = 0.83, *df* = 2, *P* = 0.66; Fig. 3B). When the HA sample was split by premorbid cognitive status, there were no significant differences in total tau in either total homogenate (*W* = 20, *P* = 0.11) or synaptoneurosome preparations (*W* = 9, *P* = 0.99). p-tau-181 was detected in total brain homogenate and in synaptic fractions across all conditions. In total homogenate preparations, there were no significant differences in p-tau181 across the three groups (Fig. 3D; *H* = 5.59, *df* = 2, *P = 0*.06), nor in synaptoneurosome preparations (*H* = 5.38, *df* = 2, *P* = 0.07; Fig. 3E). There were no significant differences in p-tau181 between the HA sub-groups of lifetime cognitive resilience or lifetime cognitive decline in either total homogenate (*W* = 6, *P* = 0.26) or synaptoneurosome preparations (*W* = 10, *P* = 0.90).

**Figure 4:**
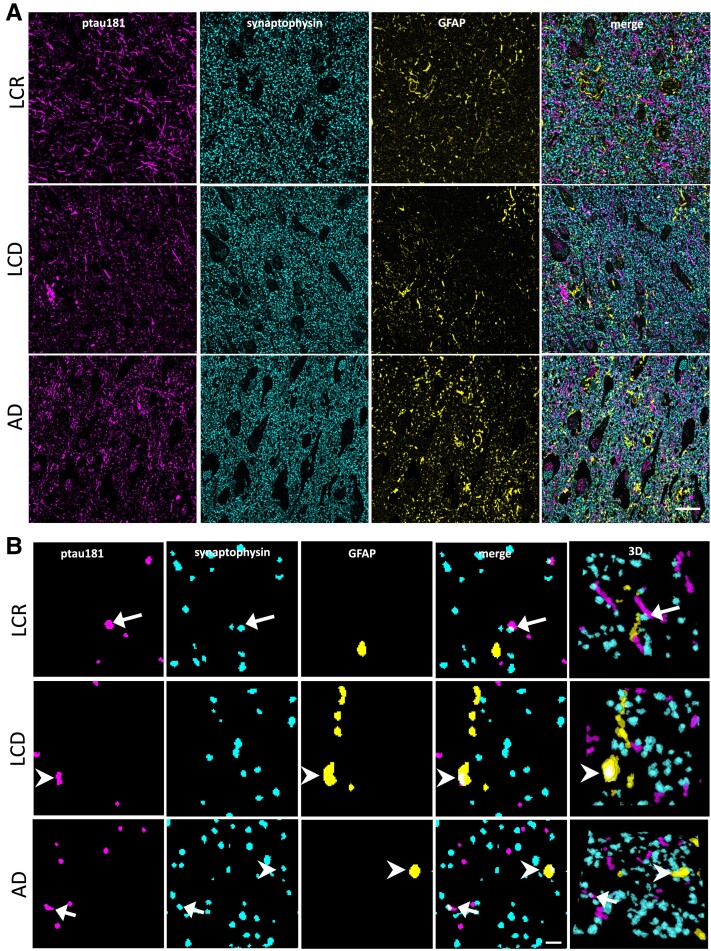
**Array tomography imaging of p-tau181, PSD95, and synaptophysin.** (**A**) Maximum intensity projections of five serial sections shows p-tau181 (magenta), synaptophysin (cyan) and GFAP (magenta) in temporal cortex of cognitively healthy ageing participant in LBC1936 (LCR, *n* = 1), an LBC1936 participant with cognitive decline (LCD, *n* = 1), and a person who died with Alzheimer’s disease (AD, *n* = 1). All groups showed p-tau181 staining in a pattern resembling neurites with some punctate staining that colocalized with synapses or astrocytes. (**B**) Image stacks were thresholded and single-section noise removed. Single sections (left) and 3D reconstructions of 10 consecutive sections (far right panel) show p-tau181 colocalization with synapses (arrows) and with GFAP-positive astrocytes (arrowheads). Scale bars represent 20 μm (**A**), 2 μm (**B**).

**Figure 5:**
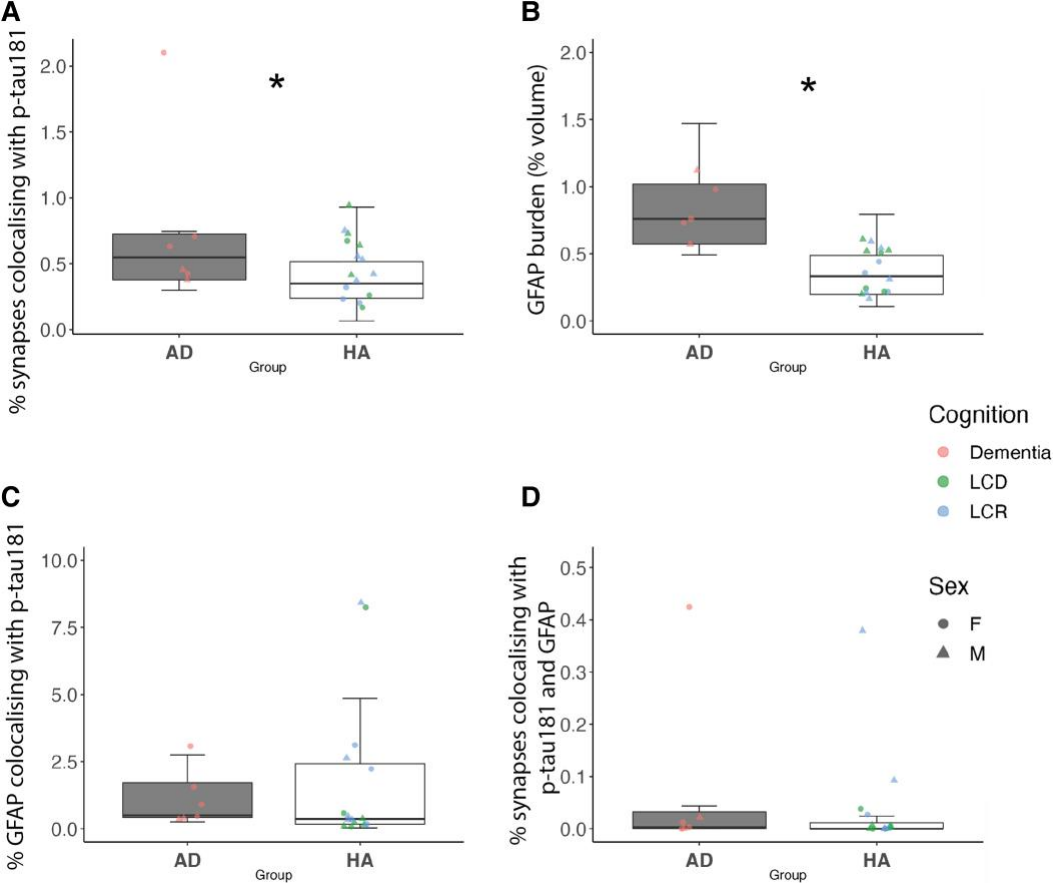
**Boxplots showing quantification of array tomography image stacks.** Data were analysed using linear mixed effects models, with cohort, sex, age and *APOE* genotype entered as fixed-effects, and case number as a random effect. Percentage of synapses colocalizing with p-tau181 between (**A**) AD and HA cases showing a significant difference between groups (*F* (1, 15.86) = 5.59, *P* = 0.03) and no significant difference between LCD and LCR cases (*F* (1, 17.23) = 1.24, *P* = 0.28). (**B**) The burden of GFAP was significantly higher in AD compared to HA cases (*F* (1, 16.4) = 14.7, *P* < 0.01) and in LCD compared to LCR cases (*F* (1, 27.59) = 7.77, *P* < 0.01). (**C**) The percentage of GFAP colocalizing with p-tau181 was not significant between AD and HA cases (*F* (1, 16.84) = 0.24, *P* = 0.63) but was significantly higher in LCR cases relative to LCD (*F* (1, 24.54) = 5.41, *P* = 0.03). (**D**) The percentage of synapses colocalizing with both p-tau181 and GFAP was not significantly different between AD and HA cases (*F* (1, 12.38) = 3.27 *P* = 0.09) or between LCD and LCR cases (*F* (1, 11.01) = 0.05, *P* = 0.83). Each data-point represents one participant. AD = Alzheimer’s disease (*n* = 7); HA = healthy agers (*n* = 16); LCD = lifetime cognitive decline (*n* = 7); LCR = lifetime cognitive resilience (*n* = 8). Significant group difference represented by *, where *P* < 0.05.

While western blots can detect relatively large changes in levels of proteins between groups, they cannot determine detailed subcellular localization or presence of proteins within individual synapses. For this more detailed level of analysis, we used high resolution array tomography imaging to examine whether p-tau181 is detectable within individual synaptic terminals and/or GFAP-positive astrocytes in human brain tissue. We observed p-tau181 in temporal cortex samples from both HA and AD brain tissue (Fig. 4). p-tau-181 immunostaining reveals staining in a neuritic pattern with some colocalization with both presynapses and astrocytes (Fig. 4). There was a significant increase in the percentage of presynaptic terminals (labelled with synaptophysin), which contain p-tau181 in AD cases (*F* (1, 15.86) = 5.59, *P* = 0.03; see Fig. 5A). Consistent with the western blot data, within the HA group, there were no significant differences between LCR and LCD cases (*F* (1, 17.23) = 1.24, *P* = 0.28; Fig. 5B). Significantly more grey matter volume was occupied by GFAP-positive astrocytes in AD cases relative to HA cases (*F* (1, 16.4) = 14.7, *P* < 0.01; Fig. 5C). GFAP burden was also significantly higher in LCD relative to LCR cases (*F* (1, 27.59) = 7.77, *P* < 0.01; Fig. 5D). The colocalization of GFAP and p-tau181 was calculated to examine whether p-tau181 accumulates in GFAP-positive astrocytes. While colocalization was observed in both groups (see Fig. 4B), there were no significant differences in colocalization percentage between AD and HA cases (*F* (1, 16.84) = 0.24, *P* = 0.63; Fig. 5E). There was a significant difference between LCR and LCD cases, with LCR cases having a higher percentage of colocalization of GFAP and p-tau181 (*F* (1, 24.54) = 5.41, *P = 0*.03; Fig. 5F). Finally, we examined the percentage of synapses colocalizing with both GFAP and p-tau181 to examine whether astrocytes may be ingesting tau-containing synapses. There were no significant differences between AD and HA cases (*F* (1, 12.38) = 3.27 *P* = 0.09; Fig. 5G), or between LCR and LCD cases (*F* (1, 11.01) = 0.05, *P* = 0.83; Fig. 5H).

**Figure 6:**
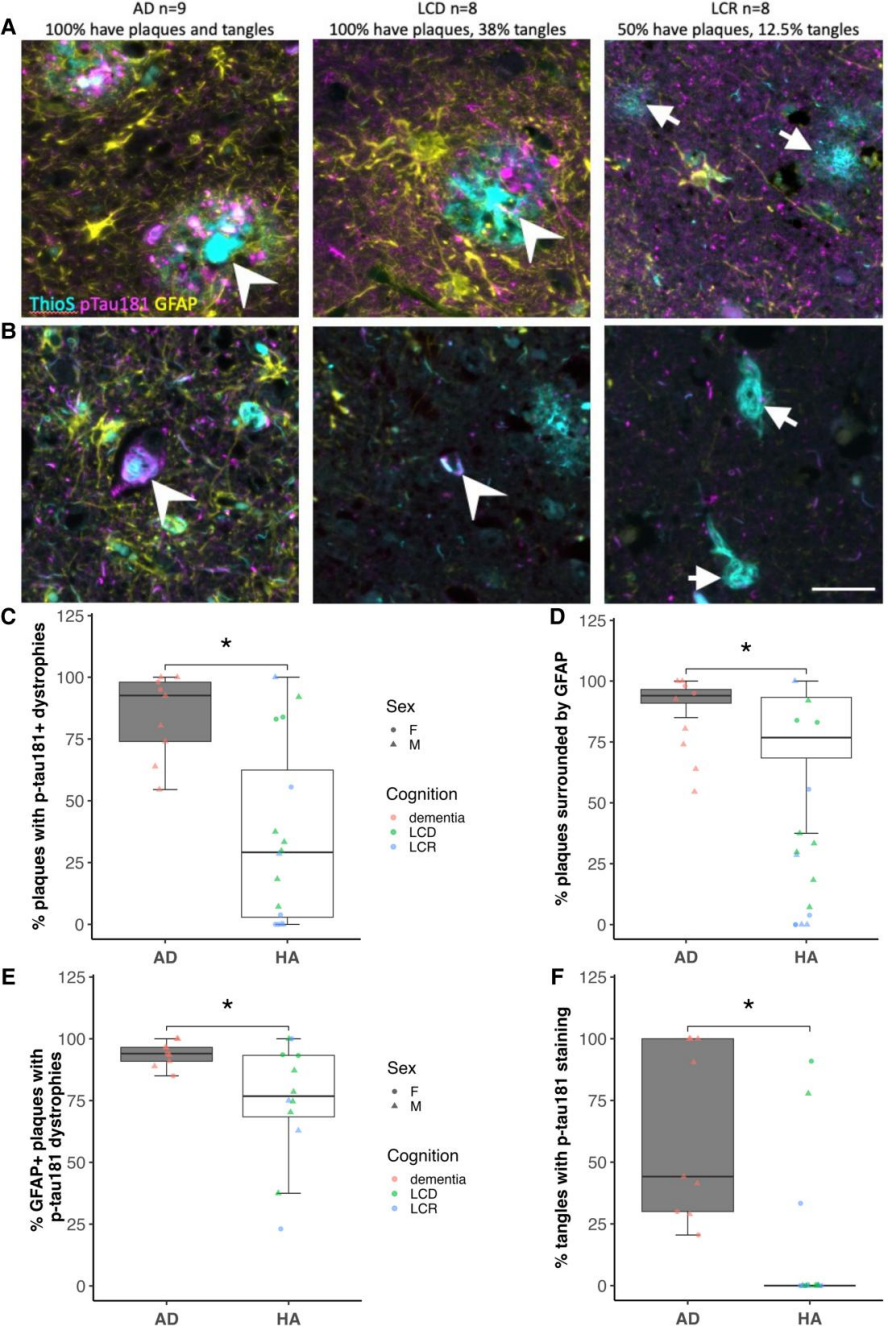
**p-tau181 is located in dystrophic neurites around some plaques and in some tangles.** To test whether plaques (ThioS, cyan) were surrounded by p-tau181-positive dystrophic neurites (magenta) or astrogliosis (GFAP, yellow), immunofluorescence was used in AD cases and healthy ageing (HA) cases with either lifetime cognitive resilience (LCR) or lifetime cognitive decline (LCD). Plaques and tangles were present in all AD cases; all LCR cases had plaques and 50% of LCD cases had plaques. Examples of plaques positive for both p-tau181 dystrophic neurites and a ‘shell’ of GFAP are indicated with chevrons and plaques without p-tau181 dystrophies or a GFAP shell indicated with arrows (**A**). Neurofibrillary tangles were also stained with ThioS (cyan, B). All AD cases had tangles, 38% of LCD cases had tangles and 12.5% of LCR cases had tangles. Examples of tangles containing p-tau181 are indicated with chevrons and tangles without p-tau181 indicated with arrows in **B**. Quantification of the percentage of plaques with p-tau181-positive dystrophies (**C**) shows a significantly higher proportion in AD cases than healthy agers [linear model with disease, sex and *APOE4* status as independent variables, effect of disease, *F* (1,17) = 8.34, *p* = 0.01, no effect of sex or *APOE4*]. A higher percentage of plaques in AD cases were also surrounded by GFAP-positive astrocytes than plaques in healthy agers [**D**, effect of disease *F* (1,17) = 5.55, *P* = 0.03, no effect of sex or *APOE4*]. Plaques were surrounded by both GFAP-positive astrocytes and p-tau181 dystrophies more in AD than HA cases [*F* (1,15) = 7.64, *P* = 0.01, **E**]. Although tangles are rare in healthy agers, there was a significant reduction in the proportion of p-tau181-positive tangles in HA compared to AD cases [**F**, *F* (1,17) = 15.49, *P* < 0.01]. Each data-point represents one participant. Data were analysed using linear regression models. Scale bar represents 20 μm. AD = Alzheimer’s disease (*n* = 9); HA = healthy agers (*n* = 16); LCD = lifetime cognitive decline (*n* = 8); LCR = lifetime cognitive resilience (*n* = 8). Significant group difference represented by *, where *P* < 0.05.

Next, we examined whether p-tau181 is detectable within dystrophic neurites around plaques in temporal cortex and whether there was an association with plaque-associated astrogliosis (Supplementary Fig. 2). We observed plaques in all nine AD cases examined. In HA, there were more plaques in people with lifetime cognitive decline (all cases with LCD had plaques) than in people with lifetime cognitive resilience (four of the eight LCR cases had plaques). We observed plaques surrounded by p-tau181-positive dystrophic neurites in both HA and AD brain tissue (Fig. 6). There was a significant increase in the percentage of plaques with p-tau181 dystrophies in AD cases relative to HA cases (Fig. 6). GFAP accumulation was observed around plaques as expected with a higher percentage of plaques surrounded by GFAP in AD than control cases (Fig. 6). The majority of GFAP-positive plaques were also surrounded by p-tau181-positive dystrophies in AD cases with less GFAP-positive plaques surrounded by p-tau181-positive dystrophies in healthy ageing cases (Fig. 6). Across all plaque measurements, when the HA group was split into LCR and LCD and compared to AD to assess effects of cognition, there was a significant effect of cognitive status (AD dementia, LCR and LCD), but *post-hoc* analyses show no significant difference between LCR and LCD.

In the same tissue stains, we quantified the proportion of NFTs that contain p-tau181. While all AD cases had NFTs in the temporal cortex samples, only 38% of LCD and 12.5% of LCR cases had tangles. NFTs contained p-tau181 staining more often in AD than HA (Fig. 6, Supplementary Fig. 1).

## Discussion

Here we have identified a significant association between cognitive ageing and baseline plasma p-tau181, NfL and GFAP, respectively, but not Aβ42/40. That is, individuals with elevated baseline blood markers had greater cognitive decline ∼10 years later. Further, we report a significant association between p-tau181 change and cognitive ageing, where those with a greater increase in plasma p-tau181 over ∼10 years had greater cognitive decline over the same period. These associations largely remained significant after controlling for medical comorbidities. We also found that with increasing age, *APOE* ε4 carriers showed significantly higher p-tau18 and lower levels of Aβ42/40. Associations between p-tau181/NfL/GFAP, and *g* factor decline were corrected for *APOE* status, one of the predictors of cognitive ageing in this and other samples,^109–112^ and remained significant. This highlights the unique potential value of these markers in accounting for differences in cognitive ageing among community-dwelling older adults. After controlling for a PRS for AD, associations remained significant (but effect sizes were attenuated) for plasma p-tau181 change in the total sample, and NfL change in females only. This suggests that both baseline and changes in biomarker levels may be partially regulated by the same genetic factors that are associated with AD, but that longitudinal increases are not solely due to incipient AD. In brain p-tau181, we found no significant differences in either total homogenate or synaptoneurosome preparations across HA, ML and Alzheimer’s cases. We also report evidence of the presence of p-tau181 in synapses, with a larger percentage of p-tau181 in pre-synapses in AD cases relative to HA cases. Further, in HA cases with cognitive resilience, the volume of GFAP occupied by ptau181 was significantly higher than in HA cases with cognitive decline. Examining plaque and tangle pathologies shows more plaques surrounded by p-tau181-positive dystrophic neurites and astrogliosis in AD than HA and more NFTs positive for p-tau181 in AD than HA.

Our findings suggest that plasma p-tau181 and NfL may be informative about cognitive decline in those without a diagnosis of dementia. Our findings are in agreement with two studies that reported a significant association between plasma p-tau181 and cognitive decline over 4 years in cognitively unimpaired participants.^18,22^ Much of the existing literature, however, failed to find a significant association between plasma p-tau181 and cognitive decline in sub-group analyses of cognitively unimpaired individuals alone. Our findings also support previous work that reports a significant association between plasma NfL and longitudinal cognitive decline between 6 and 11 years.^51,56,57^ Previous work has reported associations between plasma GFAP and cognitive functioning in cognitively unimpaired participants,^75,76^ albeit in the same cohort and with a relatively short follow-up time of 12 months. To our knowledge, our study is the first to replicate this finding in another cohort with a follow-up time of ∼10 years, although this association was not significant after controlling for a PRS for AD. In the current study, we used a more sensitive measure of cognitive functioning associated with ageing than much of the previous work. Furthermore, our sample has the benefit of a narrow age range, which offers a more precise insight into those transitioning the 8th to 9th decades of life.

Finally, we did not find a significant association between Aβ42/40 and cognitive functioning. Moreover, we observed a significant increase in Aβ42/40 between baseline and 10-year follow-up. This finding conflicts with much of the existing literature where studies have reported a significant inverse relationship between plasma Aβ and age,^34,113^ as well as a reduction in the Aβ42/40 ratio with increasing levels of cognitive impairment and dementia diagnoses.^30,32,34,36,39^ This finding may be partially attributable to the participant characteristics of those included in the current study. At age 11, LBC1936 participants had higher IQ scores than the average at age 11 for the whole of Scotland^96^ and are likely to have more years of education and a higher socioeconomic status (SES).^114^ Given the well-documented association between SES and overall health, as well as reports of an inverse association between years of education and cortical Aβ deposition,^115^ it is possible that the individuals’ above-average health and lifestyle factors may explain lower levels of Aβ deposition reflected by increased plasma Aβ. Additional lifestyle factors have also been reported to be associated with plasma Aβ. Poor sleep quality and efficiency, physical exercise, high cholesterol and diabetes have been reported to be associated with plasma Aβ in cognitively unimpaired older adults.^116-120^ Metabolism of Aβ outside of the brain is also thought to influence plasma Aβ levels.^121-123^ Further investigation of the association between lifestyle factors and peripheral sources of Aβ are needed to understand longitudinal changes in plasma Aβ in participants without dementia.

Our study is one of the few studies examining plasma p-tau181/NfL/GFAP and cognitive function longitudinally, and one of the first to do so in an aged cohort without dementia over an ∼10-year period. Our advanced statistical treatment of cognitive change, which was based on measurements at four occasions, allowed us to reliably estimate cognitive change, and more precisely partition the variance attributable to cognitive declines as distinct from extant differences in prior ability. Moreover, this is one of the few studies to examine the relationship between change in these markers and change in cognitive function. Taken together, this suggests that elevated p-tau181 and NfL are present during typical ageing and so may be a suitable marker for age-related cognitive decline.

Using biochemical analyses and high-resolution imaging, we observe time that p-tau-181 is found in synapses and astrocytes in human brain. To our knowledge, this is the first study to examine p-tau181 in synapses, however, previous work has reported elevations of p-tau S396/404 and S202 in AD cases SN,^83^ as well as misfolded and hyperphosphorylated tau in both AD and age-control SN.^124^ The current study shows that p-tau181 is present within synapses in both ageing and in AD. Given that abnormal tau accumulation has been associated with synaptic dysfunction,^83,125,126^ it is plausible that synaptic accumulation of p-tau181 damages synapses, which in turn can contribute to cognitive decline. Alternatively, the distribution of p-tau181 staining in healthy-appearing neurites and its presence in synaptic fractions from people who died in mid-life without neurological disease could indicate that this phospho-epitope of tau is not pathological in the brain. In this case, loss of synaptic p-tau181 would be predicted to be associated with cognitive decline or disease. Here, we did not find a difference between HA cases with pre-morbid cognitive resilience and pre-morbid cognitive decline. This sub-sample of cases is particularly small and so this could partially be explained by a lack of power. Where post-mortem data are a precious resource, especially with pre-morbid cognitive function data, we acknowledge that a larger sample size would enable more reliable detection and estimation of effect sizes. We also report significantly higher proportion of GFAP being occupied by p-tau181 in HA cases with cognitive resilience, relative to those with cognitive decline. While any mechanism behind this is purely speculative, in those with cognitive resilience, GFAP-positive astrocytes may be consuming p-tau181 at a faster rate. In those with cognitive decline, p-tau181 may not be being cleared as efficiently, leaving it at the synapse, which could in turn contribute to cognitive decline.

The association between plasma p-tau181/NfL/GFAP and cognitive decline in typical ageing suggests they may be suitable biomarkers for age-related cognitive decline. With further confirmation in large cohorts, a model using a panel of blood biomarkers could be developed to be a powerful predictor of people at risk for cognitive decline. This could become an economical and time-efficient screening tool for the evaluation of individuals who are most at-risk of cognitive decline, and thus are most eligible for clinical trials. Our results suggest that baseline p-tau181 and change in p-tau181 have similar magnitudes of effect of cognitive decline. Looking at the standardized coefficients in the current study, there is an increase in the steepness of 0.17 SDs in cognitive function decline for every 1 SD increase of baseline p-tau181. Further, there is an increase in the steepness of 0.21 SDs in cognitive function decline for every 1 SD increase in p-tau181 over time. Our measure of change was statistically independent of baseline p-tau181, suggesting both measures are important for predicting cognitive decline. That is, those with higher baseline p-tau181 and a steeper increase over time (irrespective of baseline) are also those with steeper cognitive decline.

Our data examining p-tau181 in dystrophic neurites around plaques provide an interesting potential explanation for the specificity of p-tau blood biomarkers for AD over primary tauopathies. Due to the disruption of membrane integrity reported in dystrophic neurites, these plaque-associated dystrophies could be a source of p-tau181 leakage into biofluids. While we did not see a significant difference between the percentage of plaques surrounded by p-tau181-positive dystrophic neurites in LCR versus LCD subjects, there were substantially fewer plaques present in the LCR subjects. Thus, the increase in p-tau181 observed in blood in people with cognitive decline could reflect at least in part an increase in plaques with surrounding dystrophic neurites.

This study is not without limitations. Firstly, the sample sizes of the post-mortem studies were relatively small. This meant the sub-groups split by pre-morbid cognition consisted of 8 or fewer cases per group, which may be under-powered to detect true effects. Further, participants were selected for analyses if complete cognitive test and plasma samples were available at ages 72 and 82 to maximize the power of our longitudinal analyses. This could lead to a selection bias impacting results as we reported significant differences in education, age 11 IQ and *g* factor change between those included and those not included. However, prior work in this cohort indicates that the studies’ initial selection bias and subsequent pattern of attrition skews our design toward healthier and more-educated individuals, and that our results therefore likely underestimate effects present at the population level.^96,127^ Furthermore, it is unknown when plasma p-tau181/NfL/GFAP increases begin during the lifetime. The current sample comprised individuals in later-life. Future longitudinal research examining these markers from mid-life onwards could be useful in understanding when increases begin and whether cognitive decline can be predicted at an earlier age. Finally, LBC1936 participants with plasma data available were excluded if they had a diagnosis of dementia; however, this does not guarantee that the sample is ‘typically ageing’ and the presence of early neurodegenerative processes cannot be ruled out.

It will also be important to investigate differences, which may affect the clinical implementation of plasma biomarkers, such as sex and ethnicity differences, as well as lifestyle factors associated with p-tau levels. For example, cognitive impairment is associated with smaller changes in CSF tau in an African-American sample relative to a Caucasian sample.^128^ Furthermore, more studies are needed to compare plasma markers as a biomarker with more established biomarkers such as tau PET, MRI measures and CSF tau. Future work would benefit from examining correlations between brain p-tau181 and plasma p-tau181, which was not possible in the current study. Finally, the investigation of p-tau phosphorylated at other sites should also be investigated; plasma p-tau231 and p-tau217 have both been investigated as promising biomarkers associated with cognitive function.^129-132^

The current study provides the first evidence in an exclusively older-age sample with narrow age-range that baseline and change in plasma p-tau181/NfL are associated with subsequent cognitive decline between 72 and 82 years, beyond *APOE* status and in the absence of dementia. We also report evidence of p-tau181 accumulation in synapses and astrocytes, which may be associated with cognition. Overall, these findings suggest p-tau181 and NfL may be useful biomarkers for predicting age-related cognitive decline and suggest mechanisms involving the decreased astrocytic clearance of ptau-181 may result in its synaptic accumulation.

## Supplementary Material

fcad113_Supplementary_Data

## Abbreviations

Aβ =: amyloid-beta
APOE =: apolipoprotein E
AT =: array tomography
CI =: confidence intervals
CV =: coefficient of variation
GFAP =: glial fibrillary acidic protein
HA =: healthy agers
IQ =: intelligence quotient
LBC =: Lothian birth cohort
LCD =: lifetime cognitive decline
LCR =: lifetime cognitive resilience
M =: mean
MCI =: mild cognitive impairment
MHT =: Moray House Test Number 12
ML =: mid-life
MMSE =: mini-mental state exam
NfL =: neurofilament-light
PMI =: post-mortem interval
p-tau =: phosphorylated tau
SCD =: subjective cognitive decline
SES =: socioeconomic status
SD =: standard deviation
SIMOA =: single molecule array
SN =: synaptoneurosome
TH =: total homogenate
