# Supplementary material for: Predictive blood biomarkers and brain changes associated with age-related cognitive decline
Source: Brain Commun. 2023 Apr 6;5(3):fcad113. doi: 10.1093/braincomms/fcad113 (PMC10167767; doi:10.1093/braincomms/fcad113)
Supplement: fcad113_Supplementary_Data [file fcad113_supplementary_data.pdf]

Supplementary data for Saunders et al: Predictive blood biomarkers and brain changes associated with age-related cognitive decline

A B

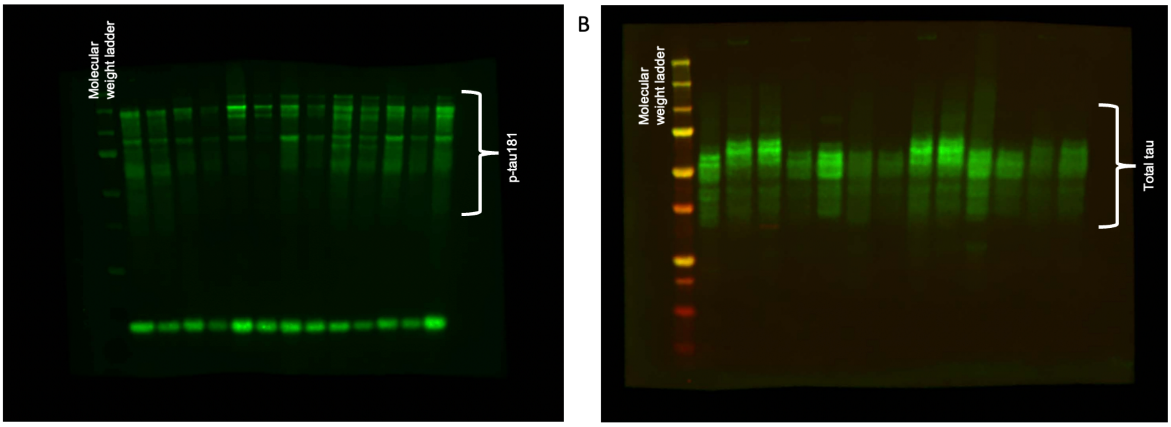

Supplementary figure 1 (related to figure 3): (A) Representative un-cropped western blot of p-tau181 across Alzheimer's cases, mid-life controls, and healthy agers in total homogenate and synaptoneurosomes (150kDa – 50kDa). Band ~ 17 kDa shows neurogranin band from a separate study. (B) Representative western blot of total tau across Alzheimer's cases, mid-life controls, and healthy agers in total homogenate and synaptoneurosomes (75 kDa – 37 KDa).

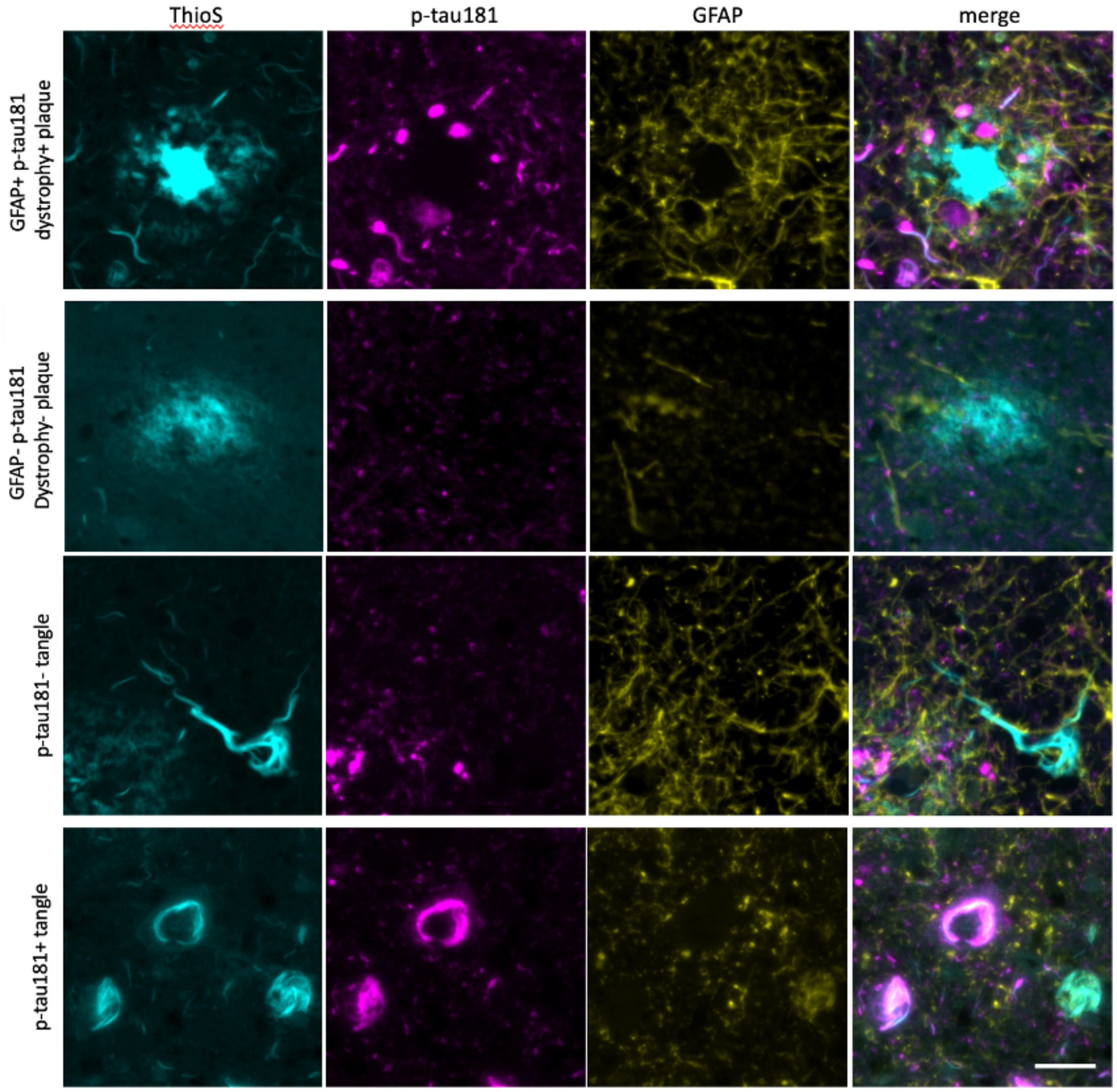

Supplementary figure 2 (related to figure 6): Representative images of each channel and merged images demonstrating classification of plaques as p-tau181 dystrophy or GFAP positive or negative and tangles as p-tau181 positive or negative. Thioflavin S was used to label plaques and tangles (cyan) and immunofluorescence was used to label p-tau181 (magenta) and GFAP (yellow). Scale bar represents 20 μm.

	p-tau181		*NfL*		*GFAP*		Aβ1-40		Aβ1-42		Aβ42/40	
Comorbidity	*β* (95% CI)	*P*	*β* (95% CI)	*P*	*β* (95% CI)	*P*	*β* (95% CI)	*P*	*β* (95% CI)	*P*	*β* (95% CI)	*P*
Diabetes	0.04 (-0.53 – 1.04)	0.60	0.01(-2.37 – 3.37)	0.91	-0.09 (-31.34 – 15.25)	0.22	0.02 (-9.32 – 11.71)	0.79	-0.05 (-1.17 – 1.08)	0.45	-0.12 (-0.02 – 0.01)	0.11
Diastolic blood pressure (sitting)	0.14 (0.01 – 0.05)	**0.04***	-0.11 (-0.13 – 0.01)	0.14	-0.03 (-0.73 – 0.43)	0.71	-0.06 (-0.39 – 0.09)	0.42	-0.08 (-0.03 – 0.01)	0.29	0.01 (-0.01 – 0.01)	0.85
Diastolic blood pressure (standing)	0.17 (0.01 – 0.05)	**0.01***	-0.11 (-0.13 – 0.01)	0.16	-0.03 (-0.79 – 0.39)	0.71	-0.06 (-0.36 – 0.08)	0.39	-0.07 (-0.03 – 0.01)	0.34	0.01 (-0.01 – 0.01)	0.91
Systolic blood pressure (sitting)	0.17 (0.01 – 0.03)	**0.01***	0.04 (-0.02 – 0.06)	0.59	0.06 (-0.25 – 0.40)	0.41	-0.07 (-0.19 – 0.05)	0.36	-0.10 (-0.02 – 0.01)	0.17	-0.04 (-0.01 – 0.01)	0.59
Systolic blood pressure (standing)	0.14 (0.01 – 0.02)	**0.05***	-0.01 (-0.04 – 0.05)	0.88	0.03 (-0.35 – 0.32)	0.72	-0.08 (-0.17 – 0.04)	0.30	-0.11 (-0.02 – 0.01)	0.14	-0.08 (-- 0.01 – 0.01)	0.29
High cholesterol (self-reported)	-0.01 (-0.39 – 0.34)	0.94	-0.09 (-2.23 – 0.36)	0.23	0.10 (-4.52 – 16.55)	0.17	0.03 (-3.28 – 5.18)	0.64	0.08 (-0.07 – 0.72)	0.25	0.14 (0.01 – 0.01)	0.06
HDL cholesterol (mmol/L)	0.09 (-0.22 – 0.70)	0.21	0.03 (-1.17 – 1.82)	0.70	0.05 (-7.34 – 18.14)	0.49	-0.18 (-13.58 - - 2.24)	**0.02***	-0.16 (-1.04 - - 0.04)	**0.04***	0.09 (-0.01 – 0.01)	0.26
LDL cholesterol (mmol/L)	0.01 (-0.15 – 0.21)	0.85	-0.13 (-1.40 – 0.05)	0.09	-0.04 (-6.61 – 4.35)	0.59	-0.07 (-3.67 – 0.33)	0.36	-0.12 (-0.37 – 0.01)	0.12	-0.07 (-0.01 – 0.01)	0.40
Cardiovascular disease	-0.04 (-0.57 – 0.34)	0.59	-0.04 (-1.77 – 1.92)	0.58	-0.10 (-20.49 – 7.40)	0.20	-0.01 (-5.32 – 5.66)	0.99	0.02 (-0.45 – 0.51)	0.77	0.05 (-0.01 – 0.01)	0.53
HADS Anxiety score	0.08 (-0.03 – 0.10)	0.27	0.01 (-0.21 – 0.25)	0.95	0.07 (-0.85 – 3.10)	0.33	-0.06 (-0.98 – 0.50)	0.44	-0.01 (-0.09 – 0.05)	0.86	0.01 (-0.01 – 0.01)	0.90
HADS Depression score	0.06 (-0.05 – 0.13)	0.36	0.01 (-0.35 – 0.31)	0.95	0.14 (-0.35 – 5.57)	0.06	-0.11 (-1.67 – 0.68)	0.13	-0.03 (-0.13 – 0.10)	0.72	0.01 (-0.01 – 0.01)	0.97
BMI	0.02 (-0.02 – 0.05)	0.78	-0.17 (-0.36 - - 0.05)	**0.02***	-0.09 (-2.01 – 0.88)	0.22	-0.03 (-0.52 – 0.64)	0.65	0.08 (-0.01 – 0.09)	0.30	0.11 (-0.01 – 0.01)	0.15

Supplementary Table 1: Association between baseline comorbidities and baseline plasma markers Models adjusted for baseline age and sex; * $P < 0.05$

	Model 1						Model 2					
	Females		*Males*		*Total sample*		*Females*		*Males*		*Total sample*	
Wave 2	*β* (95% CI)	*P*	*β* (95% CI)	*P*	*β* (95% CI)	*P*	*β* (95% CI)	*P*	*β* (95% CI)	*P*	*β* (95% CI)	*P*
p-tau181	-0.22 (-0.08, -0.01)	**0.04***	-0.08 (-0.05, 0.02)	0.47	-0.14 (-0.06, -0.01)	0.06	-0.23 (-0.09, -0.01)	0.05	-0.08 (-0.06, 0.03)	0.51	-0.14 (-0.06, 0.01)	0.11
NfL	-0.10 (-0.01, 0.01)	0.35	-0.15 (-0.02, 0.01)	0.17	-0.12 (-0.01, 0.01)	0.10	-0.13 (-0.01, 0.01)	0.27	-0.15 (-0.02, 0.01)	0.20	-0.13 (-0.02, 0.01)	0.11
GFAP	-0.05 (-0.01, 0.01)	0.66	-0.18 (-0.01, 0.01)	0.11	-0.12 (-0.01, 0.01)	0.12	-0.05 (-0.01, 0.01)	0.69	-0.18 (-0.01, 0.01)	0.12	-0.11 (-0.01, 0.01)	0.18
Aβ1-40	0.10 (-0.01, 0.01)	0.34	-0.14 (-0.01, 0.01)	0.20	-0.01 (-0.01, 0.01)	0.86	0.06 (-0.01, 0.01)	0.65	-0.13 (-0.01, 0.01)	0.29	-0.03 (-0.01, 0.01)	0.67
Aβ1-42	0.14 (-0.01, 0.05)	0.18	-0.04 (-0.05, 0.03)	0.70	0.04 (-0.03, 0.03)	0.57	0.13 (-0.02, 0.05)	0.31	-0.04 (-0.06, 0.04)	0.73	0.04 (-0.03, 0.03)	0.66
Aβ42/40	0.10 (-1.61, 4.83)	0.37	0.15 (-2.22, 5.48)	0.18	0.12 (-0.67, 4.18)	0.10	0.10 (-1.84, 5.25)	0.39	0.14 (-3.49, 6.43)	0.26	0.13 (-0.62, 4.48)	0.12
	Females		*Males*		*Total sample*		*Females*		*Males*		*Total sample*	
Change between Wave 2 and Wave 5	*β* (95% CI)	*P*	*β* (95% CI)	*P*	*β* (95% CI)	*P*	*β* (95% CI)	*P*	*β* (95% CI)	*P*	*β* (95% CI)	*P*
p-tau181	-0.17 (-0.09, 0.01)	0.10	-0.21 (-0.09, -0.01)	0.06	-0.19 (-0.07, -0.02)	**< 0.01***	-0.15 (-0.09, 0.01)	0.19	-0.19 (-0.09, 0.01)	0.11	-0.18 (-0.07, -0.01)	**0.03***
NfL	-0.24 (-0.09, 0.01)	**0.02***	0.02 (-0.05, 0.06)	0.88	-0.11 (-0.06, 0.02)	0.16	-0.26 (-0.11, 0.01)	**0.03***	0.05 (-0.04, 0.07)	0.70	-0.08 (-0.06, 0.02)	0.35
GFAP	-0.19 (-0.08, 0.01)	0.09	-0.04 (-0.07, 0.05)	0.73	-0.12 (-0.06, 0.01)	0.13	-0.21 (-0.08, 0.01)	0.08	-0.02 (-0.06, 0.06)	0.86	-0.13 (-0.06, 0.01)	0.12
Aβ1-40	-0.04 (-0.06, 0.03)	0.73 (-0.02, 0.09)	0.12	0.31	0.04 (-0.03, 0.05)	0.64	-0.04 (-0.07, 0.04)	0.73	0.12 (-0.02, 0.10)	0.32	0.03 (-0.03, 0.05)	0.72
Aβ1-42	-0.16 (-0.09, 0.02)	0.15	0.22 (-0.01, 0.13)	0.06	0.03 (-0.04, 0.05)	0.75	-0.15 (-0.09, 0.03)	0.21	0.21 (-0.01, 0.13)	0.07	0.03 (-0.04, 0.06)	0.67
Aβ42/40	-0.18 (-0.08, 0.02)	0.10	0.01 (-0.09, 0.09)	0.93	-0.10 (-0.07, 0.02)	0.20	-0.17 (-0.07, 0.02)	0.17	0.03 (-0.08, 0.11)	0.80	-0.08 (-0.06, 0.03)	0.36

Supplementary Table 2: Association of plasma markers with *g* factor decline after controlling for Alzheimer's disease (AD) Polygenic Risk Score (PRS)

Model 1: adjusted for age, sex, AD PRS

Model 2: adjusted for age, sex, *APOE* status, years of education, age 11 IQ, AD PRS

*$P < 0.05$
